# The Effects of Nitrogen Application and Varietal Variation on the Product Quality and *In Vitro* Bioaccessibility of Bioactive Compounds of Baby Spinach Varieties Grown in a Soilless Growth Medium

**DOI:** 10.3390/foods13172667

**Published:** 2024-08-24

**Authors:** Nhlanzeko Mbalenhle Bhengu, Sephora Mutombo Mianda, Martin Makgose Maboko, Dharini Sivakumar

**Affiliations:** 1 Phytochemical Food Network Group, Department of Crop Sciences, Tshwane University of Technology, Pretoria 0183, South Africa or nhlaxbhengu@gmail.com (N.M.B.); sephoramianda@gmail.com (S.M.M.); mabokom@tut.ac.za (M.M.M.); 2 Queensland Alliance for Agriculture and Food Innovation, Centre for Food Science and Nutrition, The University of Queensland, St Lucia, QLD 4069, Australia

**Keywords:** morphological parameters, carotenoids, phenolic acids, gastrointestinal digestion

## Abstract

Baby spinach is becoming increasingly popular as a salad ingredient and needs high fertiliser rates to grow well and attain higher-quality leaves (dark green leaves). Chemical fertilisers, especially nitrogen (N), boost yields. There are many risks associated with nitrogen fertilisation. Additionally, spinach contains phenolic compounds and carotenoids. Nitrogen fertilisation affects growth, development, yield and metabolites. This study examined the impact of lower concentrations of N (0, 30, 60, 90, 120, 150 mg/L) on yield and colour properties [light intensity (L*) colour coordinates, unique for green colour (a*) and yellow colour (b*)], as well as the impact of varying N concentrations on the total phenolic content and p-coumaric acid, quercetin, ferulic acid, kaempferol, lutein, zeaxanthin, β-carotene and antioxidant activities in the baby spinach varieties ‘Acadia’, ‘Crosstrek’ and ‘Traverse’, and it was established that N fertilisation improves phytochemical bioaccessibility and antioxidant activity. In a split strip plot design, three baby spinach varieties were treated with different N concentrations, including 0, 30, 60, 90, 120 and 150 mg/L. For 40 days, three baby spinach varieties were grown on soilless Mikskaar Professional substrate 300. During both seasons, ’Crosstrek’ had the highest fresh mass (921.4 g/m^2^, 856.3 g/m^2^) at 120 mg/L N, while ‘Traverse’ had the highest fresh mass at 554.8 g/m^2^ and at 564.3 g/m^2^ at 90 mg/L N and did not differ significantly from 90 to 150 mg/L N during either season. During both seasons, ‘Acadia’ at 90 mg/L N increased fresh mass to 599 g/m^2^ and 557.9 g/m^2^. The variety × N supply interaction significantly affected the leaf colour; chlorophyll content across seasons; the levels of bioactive compounds, p-coumaric acid, quercetin, ferulic acid, kaempferol, lutein, zeaxanthin and β-carotene in spinach varieties; the *in vitro* bioaccessibility; and the antioxidant activity. Varietal differences influenced the bioaccessibility of phenolic compounds and carotenoid components. The appropriate N levels can be used during plant cultivation to optimise the bioaccessibility of this spinach variety. Thus, fertilising ‘Traverse’ with 90 mg/N mL increased the *in vitro* bioaccessibility of β-carotene (35.2%), p-coumaric acid (7.13%), quercetin (8.29%) and ferulic acid (1.92%) without compromising the yield.

## 1. Introduction

Nitrogen (N) application improves foliage growth (yields) and phytochemical content in lettuce leaves [1]. However, N fertilisers are costly and environmentally damaging fertilisers. The application of a high level of nitrogen can cause nitrate levels to increase in spinach tissues, which can be harmful to consumers [2]. The European Food Safety Authority (EFSA) estimates that vegetables and fruits contribute 50–75% of dietary nitrates [3]. The EU Scientific Committee for Food (SCF) and Joint FAO/WHO Expert Committee on Food Additives (JECFA) have set an acceptable daily intake (ADI) of 0–3 mg/kg body weight for nitrate ion [4]. Thus, it is crucial to avoid an oversupply of N fertilisers without limiting the spinach genotype yield potential [5]. Choosing low-accumulating genotypes and optimising N fertilisation rates help cause minimum nitrate accumulation in vegetables [5]. 

Baby spinach (*Spinacia oleracea* L.), a member of the Amaranthaceae family, is a relatively recently developed vegetable on the market that matures in less than 40 days, from seed transplantation to being ready for consumption [6]. Spinach requires a significant amount of nitrogen to grow and maintain its deep green colour. Inorganic fertilisers containing N (>150 kg N/ha) improve plant growth [7]. Furthermore, N is essential for producing secondary metabolites like phenols. Nitrogen deficiency increases spinach’s phenolic levels [8]. The application of N increases spinach’s carotenoid content [9]. The agricultural sector is rapidly adopting soilless culture due to its ability to reduce soil-borne pathogens, soil degradation and the loss of arable land [9]. It is also possible to manage plant nutrition using soilless culture [2].

Alternatively, N supply in soilless culture, specifically in the root zone, can affect growth attributes such as plant height and leaf size and number, as well as quality attributes, including colour, chlorophyll content and phytochemicals. Moreover, spinach has high flavonoid content (>1000 mg/kg), particularly quercetin (3,5,7,3′,4′-pentahydroxyflavone), kaempferol (3,5,7,4′-tetrahydroxyflavone) and myricetin (3,5,7,3′,4′,5′-hexahydroxyflavone), along with novel flavonoid compounds like glucuronides and acylated di- and triglycosides derived from methylated and methylened dioxy derivatives of 6-oxygenated flavonols. Additionally, spinach contains several hydroxycinnamic acids, including feruoylglucose, trans and cis isomers of p-coumaric acid and meso-tartarate derivatives of it [10]. Moreover, phenolic compounds found in spinach offer numerous health benefits, such as antioxidative, anti-inflammatory, antiproliferative, anticarcinogenic and antimutagenic properties [11]. Genotype, fertilisation, climate, soil properties and leaf maturity all play a role in spinach phenolic levels and profiles [12]. 

The bioaccessibility of phytochemicals is crucial to their potential health benefits for consumers. Bioaccessibility refers to the portion of an ingested bioactive compound in the food matrix that is available for absorption in the digestive tract, ultimately influencing its bioavailability and biological activity. This study has three objectives: (1) to evaluate the influence of different concentrations of N application, including lower concentrations < 90 mg/L (0, 30, 60, 90, 120, 150 mg/L), on the yield and colour properties; (2) to investigate the impact of different low to high N concentrations on the accumulation of phenolic and carotenoids compounds and the antioxidant activity of baby spinach; (3) to establish the link between N fertilisation and improving phytochemicals in the bioaccessibility of phytochemicals and antioxidant activity during *in vitro* digestion. This research provides evidence-based information to growers, marketers and consumers on the impact of the nitrogen fertilisation of spinach and the potential bioaccessibility of phytochemicals and antioxidant activity, while ensuring higher yields for marketing.

## 2. Materials and Methods

### 2.1. Chemicals 

All chemicals used in this study were purchased from Sigma Aldrich from Johannesburg, South Africa.

### 2.2. Experimental Site, Crop Establishment and Application of N Treatments

The experiment was conducted at the Tshwane University of Technology, Gauteng Province of South Africa (25.7322° S, 28.1619° E), in a greenhouse setting over two seasons (summer and autumn). The average night/day temperatures ranged between 15 and 29, with mean average humidity of 69.68% and mean average photosynthetic active radiation of 904 µ mol/m^2^ s (Supplementary Table S1). 

Three baby spinach varieties, ‘Acadia’, ‘Crosstrek’ and ‘Traverse’ (ENZA ZADEN, South Africa), were treated with different N concentrations including 0, 30, 60, 90, 120 and 150 mg/L in a split strip plot design, replicated four times. Seeds of the three baby spinach varieties were grown on a soilless commercial Mikskaar Professional substrate 300 (Hygrotech SA. Pty. Limited, South Africa, Pretoria) growth medium for 40 days. During this period, fertigation was carried out; 2% hygroplex plus [iron (73 g/kg); manganese (19.0 g/kg), zinc (9.5 g/kg); copper (1.5 g/kg); boron (24 g/kg); and molybdenum (2.5 g/kg)], 20% potassium dihydrogen orthophosphate (K and P), 23,6% calcium chloride, 44% magnesium sulphate, 28% potassium sulphate and 13% potassium chloride were included as the nutrients applied. Fertigation was applied at a starting volume of 250 mL and up to 1000 mL per seedling tray as the seedlings developed leaf growth when the growth medium moisture status was stated as ‘dry’.

### 2.3. Leaf Fresh Weight, Colour and Chlorophyll Content 

At harvest (after 40 days of seed sowing), 50 seedling samples per variety and N concentration were randomly selected for the analysis of yield (fresh) and quality (chlorophyll index and colour). Leaf fresh weight, colour and chlorophyll content were determined according to Mampholo et al. [13]. The chlorophyll index was measured using the chlorophyll SPAD meter (Konica Minolta Co., Ltd., Kyoto, Japan). Leaf colour (L*a*b*) was measured using the Minolta CR 400 Chroma meter (Minolta camera, Co., Osaka, Japan). Dried weights were determined as the percentage difference between the fresh weight and oven-dried sample (48 h at 65 °C), and the results were expressed as g/m^2^. 

### 2.4. In Vitro Digestion 

The *in vitro* digestion of freeze-dried raw baby spinach leaves was conducted according to the method described by Minekus et al. (2014) [14]. This procedure included three main consecutive phases of digestion: oral, gastric and intestinal digestion (Supplementary Figure S1). Samples were collected after each phase (gastric and intestinal) for analysis. These were cooled in an ice bath to end the digestion and then stored at −80 °C for further analysis. The bioaccessibility was calculated using the following equation [15]:

(1) $$\mathrm{Bioaccessibility}=\frac{B_{SI}}{B_{ND}}\times 100$$

where *B_SI_* shows the content in the intestinal fraction and *B_ND_* indicates the content in the undigested sample.

### 2.5. Extraction of Phenolic Compounds 

The samples (100 mg) were mixed with 2 mL of methanol/water (80:20), then sonicated using an ultrasonic bath (42 kHz, 135 W, Branson Ultrasonic Corporation, Buffalo, NY, USA) for 30 min. Afterward, the samples were centrifuged (HermLe Z326k, HermLe Labortechnik GmbH, Wehingen, Germany) at 1000× *g* for 10 min at 4 °C. The supernatant was collected. The process was repeated twice on the residue and the supernatants combined. The extracts were used for the analysis of total phenolic content, phenolic profiling using HPLC and antioxidant activities. 

### 2.6. Total Phenolic Content (TPC)

Total phenolic content was determined using the Folin–Ciocalteu method [16]. Briefly, 100 µL of extract (Section 2.5) was mixed with 200 µL of 10-times-diluted Folin–Ciocalteu solution and 800 µL of 7.5% Na_2_CO_3_ solution in water. The mixture was incubated for 2 h at room temperature, and then, the absorbance was measured at 765 nm using a spectrophotometer

(SPECTROstar^®^Nano BMG Labtech, Ortenberg, Germany). Gallic acid was used as a standard and the results were expressed as gallic acid equivalents (GAEs) in mg/g dry weight (dw) of sample.

### 2.7. Quantification of Phenolic Compounds 

The quantification of targeted phenolic compounds was carried out using a Shimadzu HPLC-DAD system (LC-2030C, Shimadzu Corp, Kyoto, Japan) described by Mpai et al. [17]. The phenolic extract (Section 2.5) was filtered through a 0.22 μm PTFE syringe filter (13 mm diameter), and 10 µL was injected three times into the HPLC. The separation was achieved on a Luna^®^ C18 column (4.6 × 250 mm, 5 µm, Phenomenex) with a gradient elution of solvent A (water + 0.1% formic acid) and solvent B (methanol/acetonitrile, 40:60), starting with 0% B in A to reach 10% B at 5 min, 15% B at 10 min, 25% B at 15 min, 35% B at 25 min, 100% B at 40 min and the flow rate of 0.6 mL/min. The quantification of phenolic compounds was achieved using the calibration curves constructed using pure external standards (p-coumaric acid, ferulic acid, quercetin and kaempferol). The quantified compounds are reported in µg/g.

### 2.8. Ferric Reducing Antioxidant Power (FRAP)

FRAP value was determined as described by Xiao et al. [18] without any changes, using a 96-well microplate reader (BMG LABTECH GmbH, Spectro Star Nano, Ortenberg, Germany). The FRAP working solution was prepared by mixing 2,4,6-tri(2-pyridyl)-1,3,5-triazine (TPTZ) solution (10 mmol/L), acetic acid buffer (pH 3.6) and FeCl_3_ solution (20 mmol/L) in a ratio of 1:10:1. The mixture was then incubated for at 37 °C for 15 min and used up within 1–2 h. In a 96-well plate, 180.0 µL of FRAP working solution and 10 µL of sample extract (Section 2.5) were mixed and incubated at 37 °C for 15 min in the dark before the absorbance was read at 593 nm. Trolox was used as the standard and prepared at different concentrations (0–0.1 mg/mL). The results were calculated against the calibration curve Y = 0.0048x + 0.1274 (R^2^ = 0.99) of the TROLOX standard and expressed as TEAC mM/g dw.

### 2.9. 2,2-Azino-bis-3-ethylbenzothiazoline-6-sulfonic (ABTS) Scavenging Activity

The ABTS assay was carried out using the method described by Xiao et al. [18]. Briefly, an ABTS reaction solution was prepared by mixing 5 mL of ABTS solution (7 mmol/L in acetic acid buffer pH 4.6) and 5 mL of potassium persulfate (K_2_S_2_O_8_) solution (2.45 mmol/L). The mixture was kept overnight at room temperature in the dark. Then, 5 mL of the ABTS reaction solution was diluted to 50 mL in phosphate buffer to obtain the ABTS working solution, which was kept for 30 min in the dark at room temperature. Different concentrations of the phenolic extract (Section 2.5) made from serial dilutions were made and transferred into wells of a 96-well plate. Then, 200 µL of the ABTS working solution was added. The mixtures were allowed to react in the dark for 30 min before the absorbance was read at 750 nm in a microplate reader well. The percentage of inhibition was calculated using the following formula:

Inhibition ratio (%) = (A0 − A1) × 100/A0(2)

A0 and A1 represent the ABTS assay absorbance and sample absorbance, respectively.

IC_50_ values were calculated from the regression line resulting from plotting the inhibition ratio (%) against the sample concentration, and the results were expressed as mg/mL.

### 2.10. 2,2-diphenyl-1-picrylhydrazyl (DPPH) Radical Scavenging Activity

The DPPH radical scavenging activity of samples was determined by using the method described by Xiao et al. [18] with some modifications. Briefly, different concentrations (0–10 mg/mL) were prepared from the sample extract (Section 2.5) and 100 µL of each was transferred into a microplate reader and well mixed with 200 µL of DPPH working solution and incubated in darkness to allow a reaction for 30 min before the absorbance was read. The IC_50_ values were calculated as for ABTS (2.9). The results were expressed as mg/mL.

### 2.11. Carotenoids

Carotenoid extraction was carried out according to the procedure described by Djuikwo et al. [19], with some modifications. Samples (100 mg) were homogenised with 5 mL of 95% ethanol containing 0.1% (*w*/*v*) butylated hydroxytoluene (BHT) for 10 min before saponification with 1 mL of KOH solution (20% in methanol, *w*/*v*) for 30 min while shaking. Afterward, carotenoids were extracted three times with 3 mL of hexane/dichloromethane mixture (70:30, *v*/*v*) containing 0.1% BHT. A solution of 10% NaCl (*w*/*v*) was added for phase separation before centrifugation at 3900× *g* for 5 min. The upper layer was collected, combined and evaporated under a nitrogen stream until dry. The dried extract was freshly reconstituted in methanol/MTBE (50:50, *v*/*v*) + 0.1% BHT for total carotenoid content and HPLC analysis. To determine the total carotenoid content, different concentrations (0–0.1 mg/mL) of β-carotene standard were prepared from the serial dilution of a stock solution (0.1 mg/mL). The absorbance of reconstituted extracts and standards were read at 453 nm using a spectrophotometer and the results were expressed as β-carotene equivalents in mg/g dw. The individual carotenoids were identified and quantified using HPLC (Shimadzu, LC-2030C 3D, Kyoto, Japan). The freshly reconstituted extract was filtered using a PTFE syringe filter (0.22 µm pore size, 13 mm diameter), and then, 10 µL was injected into the HPLC three times. The separation of carotenoid compounds was achieved on a InertSustain C30 column (3.0 × 250 mm, 3 µm, GL Sciences, Tsukuba, Japan) with a gradient elution of 0.1% of formic acid in methanol (solvent A) and methyl tert-butyl ether (solvent B) as follows: 0% B in A to reach 5% B at 1 min, 50% B at 37 min and back to 0% B at 38 min at a flow rate of 0.3 mL/min. Chromatograms were recorded at 460 nm. The quantification of carotenoids was achieved using calibration curves constructed with carotenoid standards, which included lutein, β-carotene and zeaxanthin.

### 2.12. Data Analysis

The data were analysed using the GenStat 18th version statistical package (VSN International, Hempstead, UK) using two-factorial analysis (three varieties and six N levels) and repeated twice. Fresh weight and colour properties were analysed with ten replicate samples in each season, while biochemical properties were analysed with five replicates per season. The mean separation for significant variation was achieved using Tukey’s at the significance level of 5%.

## 3. Results and Discussion

### 3.1. Effect of Varietal Response and N Supply Levels on Leaf Fresh Weight 

Supplementary Table S2A,B show a significant interaction between spinach varieties and N levels affecting fresh mass in both growing seasons. Moreover, we observed a positive linear trend between leaf fresh mass and N-level concentrations in the three studied spinach varieties (Supplementary Figure S2A,B). The ‘Crosstrek’ variety with an N supply of 150 and 120 mg/L showed the highest fresh leaf mass during both seasons (Season 1 and Season 2) (Table S2B). Meanwhile, vars. ‘Arcadia’ and ‘Traverse’ showed the highest leaf mass with an N supply of 90, 120 and 150 mg/L. The ‘Crosstrek’ variety showed the highest leaf mass with an N supply of 120 and 150 mg/L compared to the other two varieties during both seasons (Table S2B). These findings align with those of Thapa et al. [20], who found that low levels of N led to the lowest fresh mass in spinach. Additionally, nitrogen deficiency was found to reduce the dry weight of sorghum [21]. The decrease in fresh leaves at low N application levels may be attributed to the reduction in leaf chlorophyll and decreased photosynthetic activity [22]. This information is essential for farmers to put into practice.

### 3.2. Effect of Varietal Response and N Supply Levels on Colour

The negative a* values indicate the intensity of green colour, positive b* values describe the intensity of yellow colour and the L* value describes the lightness [13]. Variety × N supply interaction significantly (*p* < 0.001) affected the leaf colour values (L*, a* and b*) across seasons (Table S3A). Lightness (L*) was negatively correlated with N supply during both seasons (Table S3B). The highest L* value was observed at 0 mg/L of N across all three baby spinach varieties (‘Acadia’, ‘Crosstrek’ and ‘Traverse’) in both seasons with yellowish-green leaves (Table S3C). The lightness of baby spinach leaves was attributed to a reduction in chlorophyll content and photosynthetic activity [23]. The lowest L* value was observed at 150 mg/L N in ‘Acadia’, 60 and 90 mg/L N in ‘Crosstrek’ and 90 mg/L N in ‘Traverse’ during both seasons. 

The colour parameter a* was higher at 120 and 150 mg/L N for ‘Acadia’, while the N supply ranging from 30 to 150 mg/L showed a lower, non-significant a* value for the ‘Crosstrek’ variety. However, increasing N supply from 60 mg/L to 150 mg/L resulted in a non-significant decrease in a* value for the var. ‘Traverse’. 

The b* value decreased with N supply from 30 to 150 mg/L in all three baby spinach varieties. In ‘Acadia’, the b* value non-significantly reduced with the 30 to 150 mg/L N supply. Similarly, in ‘Crostrek’ and ‘Traverse’, there was a non-significant reduction in the b* value with the 60 to 150 mg/L N supply. The lack of chlorophyll in plants caused by nitrogen deficiency results in yellow leaves since chlorophyll provides plants with their green pigment [24]. Fertiliser use can be guided by identifying the nitrogen content in plant leaves based on leaf colour.

### 3.3. Effect of Varietal Response and N Supply Levels on Chlorophyll Content 

A correlation has been established between SPAD readings and chlorophyll levels by Uddling et al. [25]. There was a significant (*p* < 0.001) interaction between spinach variety and N supply which affected leaf chlorophyll content based on SPAD readings for both growing seasons (Table S4A). An increase in N supply resulted in an increase in the SPAD reading (leaf chlorophyll content). At 150 mg/L of N supply, all three spinach varieties exhibited the highest SPAD reading due to leaf chlorophyll accumulation (Table S4B). The ‘Acadia’ variety showed the highest chlorophyll content, followed by ‘Traverse’ and ‘Crosstrek’ during both seasons. Crop growth and yield are influenced by chlorophyll, a primary photosynthetic pigment in plants [26]. There is also a positive relationship between leaf chlorophyll and N supply levels (Table S4C). Nevertheless, SPAD units are affected by chlorophyll content, while the green colour is affected by varieties, stages of development, setting, weather and pests [27]. Therefore, spinach leaves with a low N supply are unlikely to appear pale.

### 3.4. Effect of Varietal Response and N Supply Levels on Bioactive Compounds and Their Bioaccessibility

#### 3.4.1. Phenols

Table 1 presents the impact of N fertilisation on three varieties of spinach concerning total phenolic content (TPC), different phenolic components and bioaccessibility during *in vitro* digestion. TPC bioaccessibility was significantly affected by spinach varietal differences and N supply levels (Table S5A). The TPC increased significantly with nitrogen (N) supply from 0 to 60 mg/L, reaching its peak at 60 mg N/mL in all three spinach varieties. However, the TPC decreased with an increasing N concentration from 90 to 150 mg/mL after reaching 60 mg N/mL in all three varieties. Notably, in ‘Traverse’, N supply of 60 mg/mL showed a significant increase in TPC levels (8.62 mg/g). 

p-Coumaric acid deceased from N0 onwards with an increasing concentration of N supply. In ‘Crosstrek’, the p-coumaric acid slightly peaked at 90 mg N/mL to 184.2 µg/g, and in ‘Traveser’, at 60 mg N/mL to 86.8 µg/mg. Additionally, ferulic acid levels peaked significantly in ‘Crosstrek’ (6020 µg/g) and ‘Traverse’ (5062 µg/g) with 60 mg/mL and 30 mg/mL N supply, respectively. Although the concentration of kaempferol showed a reducing trend from N0 to increasing concentrations of N supply, the ‘Crosstrek’ (119.4 µg/g) and Traverse (195.9 µg/g) varieties showed a significant increase in kaempferol accumulation with an N supply of 120 mg/mL. Moreover, although quercetin concentration decreased with increasing N supply, ‘Acadia’ and ‘Traverse’ showed an increase with a 60 and 90 N supply, though this increase was less than that with a 0 N supply.

In general, a high N supply adversely affects phenolic compound accumulation [28]. However, in this study, p-coumaric acid, ferulic acid, quercetin and kaempferol were the predominant phenolic compounds in all three spinach varieties at harvest (undigested samples). Due to genetic differences, their concentrations varied between varieties. In ‘Acadia’, p-coumaric acid, ferulic acid, quercetin and kaempferol decreased significantly with increasing N supply from 0 to 150 mg/mL. 

Increasing N levels in wheat resulted in lower levels of p-coumaric acid and cis-ferulic acid [29]. All three lettuce varieties accumulated chlorogenic acid in response to N supply. Following 120 kg/ha N, Multired 4 lettuce showed decreased chlorogenic acid accumulation [30]. Green lettuce varieties prefer 60 to 90 kg/ha N for flavonoid and quercetin biosynthesis, indicating a varietal response to N application [30].

According to the C/N shift theory, phenolic acids and flavonoids are synthesised using carbon-based metabolites under N deficiency [1]. Becker et al. [1] further indicated that at lower N concentrations, with regard to the activity of phenylalanine ammonia-lyase responsible for the synthesis of higher concentrations of polyphenol propanoids, there is a correlation between phenolic compounds and phenolic enzyme stimulation. It has been shown that reduced nitrogen supply induces phenylpropanoid pathway enzymes, such as phenylalanine ammonia-lyase and 4-coumarate: tobacco leaves [30] and tomato leaves [31] contain flavonoid pathway enzymes such as chalcone synthase and dihydroflavonol-4-reductase [31]. All these examples explain the increased levels of phenolic acids (p-coumaric acid, ferulic acid) and flavonoids (kaempferol, quercetin) and the lower fresh leaf weight obtained in this study.

On the contrary, nitrogen nutrition negatively impacts polyphenol biosynthesis in some plants [30]. This could be due to the fact that dilution can also lead to a decline in phenolic content. Nitrogen fertilisation also increases the biomass (dry weight) of these varieties [32,33]. Increasing biomass production reduces the concentration of phenolics when measured in phenolic weight units [33]. This dilution effect can occur even when the biosynthesis of polyphenols remains unchanged but biomass production increases. In addition to nitrogen nutrition, the accumulation of phenolics is influenced by various other nutritional, environmental, genetic and agronomic factors [34,35]. Research suggests that leafy vegetables change their phenolic profiles and their bioactivities after digestion. Accordingly, in this study, the *in vitro* digestion model was employed to investigate the bioaccessibility and activity of phytochemicals and provide a deeper exploration based on phenotypic differences in baby spinach cultivars affected by nitrogen fertilisation. 

The bioaccessibility of various phenolic components in different spinach varieties fertilised with N fertiliser is unknown, which could directly impact the health benefits for consumers. It was found that the plant genotype and N supply significantly affected TPC and phenolic components in intestinal fractions (IFs). Although at harvest (undigested), 60 mg N/L showed the highest accumulation of TPC for all three spinach varieties, ‘Acadia’ fertilised with different concentrations of N supply did not show significant (*p* < 0.001) variation in the IF and the bioaccessibility of TPC in the intestinal fraction (IF). In the ‘Crosstrek’ variety, the IF contained the highest total phenolic content (TPC) when supplied with 60, 120 and 150 mg/L of N. However, the highest percentage of TPC bioaccessibility was observed in the IF of ‘Crosstrek’ when fertilised with 150 mg/L of N. Meanwhile, the TPC was highest and significantly bioaccessible in the IF of var. ‘Traverse’ when fertilised with 60 and 150 mg/L of N. Similarly, Sęczyk, et al. [15] mentioned a significant reduction in TPC during *in vitro* digestion, regardless of the dose of N supply. Although Sęczyk et al. [15] reported a positive effect of N fertilisation on TPC for IF, our results indicate that the concentration of TPC in the IF and its bioaccessibility depends on the variety and the dose of N supply. 

In addition, a varietal interaction with N supply (at *p* < 0.001) was evident in the levels of p-coumaric acid, quercetin, kaempferol and ferulic acid in the IF (Table S5B). However, the ‘Acadia’ and ‘Crosstrek’ varieties fertilised with 90, mg/L N showed the highest percentage of bioaccessible p-coumaric acid in the IF. The percentage of bioacessibility increased by 2.7% compared to the plants supplied with 0 N. On the other hand, the ‘Traverse’ variety fertilised with 30 mg/L N displayed the highest bioaccessibility of p-coumaric acid in the IF. However, those fertilised with 30 mg/L N and 60 mg/L N displayed 5.90% and 5.16% more bioaccessibility than ‘Traverse’ supplied with no N (with N0). The values for p-coumaric acid can be taken into account as limits. It is imperative to consider them as part of a whole, as many other phenolic compounds contribute to the nutritional value and antioxidant activity of baby spinach.

The concentration of ferulic acid in the IF of ‘Acadia’ and ‘Crosstrek’ increased non-significantly with 30 mg/L to 150 mg/L N supply compared to 0 N supply. However, there was no significant difference observed in the bioaccessibility of ferulic acid from ‘Acadia’ and ‘Crosstrek’ when fertilised with 30 mg/L to 150 mg/L N supply compared to 0 N supply. On the other hand, ‘Traverse’ fertilised with a 60 mg/L N supply showed highly bioaccessible ferulic acid in the intestinal fraction like 0 N supply.

Conversely, the quercetin content was significantly high in the intestinal fraction of ‘Acadia’ when fertilised with 60 and 90 mg/L N supply. However, the percentage bioaccessibility of quercetin content in the IF of the ‘Acadia’ variety fertilised with 150 mg/L N was higher (4.26%) than that with 60 and 90 mg/L N supply. The spinach varieties ‘Crosstrek’ and ‘Traverse’, fertilised with 120 mg/L N and 30 mg/L N, respectively, showed higher bioaccessibility of quercetin in the IF. 

The concentration of kaempferol decreased in the intestinal fraction in ‘Acadia’ compared with the samples that received no N supply and 30 mg N supply; it peaked significantly with 60 mg/mL N and showed the highest bioaccessibility at 6.56% from the IF. In the IF, the concentration of kaempferol in the ‘Crosstrek’ variety, when fertilised with 60 mg/mL of N, reached 6.68 µg/g, with a bioaccessibility of 7.72%. In the intestinal fraction of the ‘Traverse’ variety fertilised with 30 mg/mL N, the kaempferol concentration was significantly higher at 25.86 µg/g. However, the bioaccessibility of kaempferol in the IF was higher for ‘Crosstrek’ fertilised with both 30 and 60 mg/mL N.

According to Sęczyk et al. [15], the statistically significant and negative effect of nitrogen fertilisation on the potential bioaccessibility of TPC was observed only for 150 mg/mL samples when compared with the unfertilised sample (N0). Our study suggests that the potential bioaccessibility of TPC and the different phenolic components varies with the plant type, variety or genotype, food matrix and N supply. Several physicochemical and biochemical components are crucial in digestion, specifically pH, ionic strength, temperature, bile salts, enzymes and bile salts. Digestion conditions affect phenolic compounds’ stability, and the release of these compounds from the food matrix is affected by interactions [35,36]. As pH changes during digestion, the protonation/deprotonation of phenolics may alter their concentration-independent antioxidant properties [35,36]. It is also possible that a change in pH due to digestion can also change the racemization of molecules and produce enantiomers that have different chemical structures and functions of polyphenols [35,36]. It is possible to increase the dietary fibre and protein content of food by fertilising it with nitrogen [35,36]. On the other hand, it was found that phenolic compounds interacted with nutritional fibres and proteins [36]. The affinity and interaction strength of phenolic compounds with food matrix components is governed by the physicochemical properties of polyphenols and food matrix components [35,36]. When the food matrix degrades and undergoes physicochemical modification, it can release phenolic compounds that were bound to it [35,36]. The decomposition of the food matrix can reveal new binding sites for polyphenols and increase their affinity [35,36]. All of these factors mentioned above could affect the bioaccessibility of phenolic compounds in the intestinal phase.

In addition, Greek oregano fertilised with 30–120 kg N ha1 (N30–N120) had a positive effect on the intestinal rosmarinic acid index, with the highest level observed with N supply at 30 kg N ha1 [14]. Similarly, the bioaccessibility of different phenolic components varied with different N supply doses and varieties (genotypes). The ‘Traverse’ variety at 60 mg/L N supply showed the highest level of bioaccessible ferulic acid in the IF. Additionally, ‘Traverse’ at 60 and 90 mg/L N supply increased the bioaccessibility of quercetin in the intestinal fraction. Also, ‘Traverse’ at 30 and 60 mg/L N supply increased the bioaccessibility of p-coumaric acid and kaempferol in the IF. 

The bioaccessibility of ferulic acid and kaempferol in the IF of vars. ‘Crosstrek’ and ‘Acadia’ were not affected by N supply. At 90 and 120 g/L N supply, vars. ‘Acadia’ and ‘Crosstrek’ demonstrated the highest bioaccessibility of p-coumaric acid. Additionally, quercetin is most bioavailable from ‘Acadia’s’ IF at 150 g/L N supply and that of var. ‘Crosstrek’ at 120 g/L N supply. Thus, our observations confirm that in addition to the differences in N supply, the variable of the plant matrix which can be related to the varietal difference also affects the bioaccessibility of phenolic compounds from the IF.

#### 3.4.2. Carotenoids

The effect of N supply levels on the total carotenoid content (TCC) and individual carotenoid compounds at harvest (undigested sample) and their bioaccessibility of three baby spinach varieties is shown in Table 2. The results showed that increasing N supply levels had a positive effect on the accumulation of TCC in baby spinach varieties at harvest (undigested samples) compared to an N0 supply. All three spinach varieties reached their maximum TCC at 150 mg/L N. These results support the findings of Hedren et al. [37] and O’Conell et al. [38] with carotenoid increase and N application. However, our results are contrary to the results of Zikalala et al. [26], who showed that N application had no significant effect on baby spinach and kale. Carrots fertilised with N between 60 and 120 kg/ha N showed a similar increase in TCC [5]. 

A clear interaction exists between variety and N supply levels for total carotenoids (at *p* < 0.001) during *in vitro* digestion (Table S6A). Furthermore, the concentration of TCC in the IF was lower than that found in the undigested samples of all three spinach varieties fertilised with different levels of nitrogen supply. Conversely, all three spinach varieties fertilised with 150 mg/L of nitrogen showed the highest TCC in the IF with the highest bioaccessibility. The study findings support the notion that excessive nitrogen doses may impede plastid synthesis and chloroplast-to-chromoplast conversion during the green stage [38,39]. An increase in carotenoids and chlorophyll in baby spinach leaves would greatly increase the contribution of dietary nutrition to the human diet [36]. The differences between plant varieties and N supply during fertilisation play a significant role in influencing the health benefits of consuming vegetables. The bioaccessibility of carotenoid in spinach was influenced by genotype, and the stability of the bioaccessible content was more critical for spinach genotype nutritional breeding [36].

*In vitro* digestion exhibited a clear interaction between variety and N supply levels at *p* < 0.001 for carotenoid components lutein, alpha-carotene and zeaxanthin (Table S6). Carotenoid components such as lutein and β-carotene concentrations in all three spinach varieties increased with increasing N supply from 30 to 150 mg/L at harvest (undigested sample) and in the intestinal fraction during *in vitro* digestion. According to Hochmuth et al. [40], carrots with 160 kg of nitrogen per ha have the highest ß-carotene levels (55 mg/kg). Our study examined the *in vitro* bioaccessibility of carotenoids, which is an aspect of N supply in vegetables that has been largely overlooked. The carotenoid components in the intestinal fraction were significantly increased with the concentration of N supply and reached the maximum at 150 mg/L N. However, the highest potential bioaccessibility of the carotenoid components from the intestinal fraction was affected by the plant genotype and nitrogen supply (*p* < 0.05). Even though the increase in N supply led to higher concentrations of carotenoids in the IF during the *in vitro* digestion of these spinach varieties, the bioaccessibility of the carotenoid components was higher from the IF at lower levels of N supply. In contrast, ‘Crosstrek’ and ‘Traverse’, which received 30 mg/L N supply, showed the greatest percentage of lutein bioaccessibility in the IF, while ‘Acadia’, which received 60 mg/L N supply, had the highest percentage of lutein bioaccessibility. The ‘Acadia’ variety, fertilised at 60 mg/L N, and ‘Traverse’ and ‘Crosstrek’, fertilised at 90 mg/L N, had the highest levels of β-carotene bioaccessibility. Similarly, Ukom et al. [41] demonstrated that N supply above 80 kg/ha N failed to increase the β-carotene concentration significantly. Carotenoids’ bioaccessibility is the proportion of carotenoids absorbed by the micelle fraction after digestion, compared to the original amount. However, most studies report that a very low proportion of carotenoids are bioaccessible during *in vitro* digestion [36]. Several factors appear to affect the results, such as protein and lipid contents in the diet, as well as bile acid. Additionally, fibres from the food matrix themselves may decrease carotenoid micellization [36]. The transfer of carotenoids to micelles is inversely proportional to their hydrophobicity [36]. Lutein is less lipophilic; therefore, it is more soluble in micelles since it has lower lipophilicity [36].

Conversely, the zeaxanthin concentration increased in all three varieties when nitrogen supplies increased at harvest. However, during *in vitro* digestion in the intestinal fraction, the concentration of zeaxanthin increased in ‘Acadia’ at 120 mg/L N, while in var. ‘Crosstrek’, it increased at 150 mg/L N, and in. ‘Traverse’, it increased at 60 mg/L N. The percentage of bioaccessibility of zeaxanthin was highest for ‘Arcadia’ fertilised with 60 mg/L N, ‘Crosstrek’ with 120 mg/L N and ‘Traverse’ with 30 and 60 mg/L N supply. β-carotene can enhance vitamin A status among the vulnerable population [42]. 

Therefore, for a true reflection of the effect of the N fertilisation of leafy vegetables on the bioaccessibility of phytochemicals such as phenols or carotenoids, it is necessary to examine the impact of N fertilisation on the bioaccessibility of individual components, rather than the accumulation of total phenolics and carotenoids. However, our study demonstrated that simply increasing the N supply may not produce significant results concerning the bioaccessibility of different carotenoid components. We observed the higher bioaccessibility of β-carotene, lutein and zeaxanthin in the intestinal fraction of the ‘Arcadia’, ‘Crosstrek’ and ‘Traverse’ varieties fertilised at or less than 90 mg/L of N in our study. Sęczyk, et al. [15] showed that a comparatively low N supply of 30 kg/ha strongly enhanced the bioaccessibility of phytochemicals in Greek oregano. Further, it should be noted that carotenoid accumulation is also affected by other factors besides nitrogen application, including nutrition, the environment, genetics and agronomy [43,44,45]. As a result of our observations, we can suggest that even though N application has a significant impact on the carotenoid accumulation in green leafy vegetables like spinach, the varietal difference also has the strongest effect on carotenoid bioaccessibility, with the release of carotenoids from its food matrix being the major limiting factor.

#### 3.4.3. Varietal Variation and Nitrogen Application on the Antioxidant Activity and Bioaccessibility

Spinach variety and N supply levels’ interaction (at *p* < 0.001) is evident in the IF, which affects antioxidant power (FRAP) and scavenging activities (DPPH and ABTS) (Table S7). The effects of N application on the antioxidant activity and bioaccessibilty of three baby spinach varieties are presented in Table 3. For freshly harvested undigested samples, the three baby spinach varieties exhibited different responses of ferric reducing antioxidant power (FRAP) to the N application. The ‘Acadia’ variety had the highest FRAP when supplied with 90 mg/L N and 120 mg/L N, while ‘Crosstrek’ had the highest FRAP at 120 and 150 mg/L N supply. The ‘Traverse’ variety showed the highest antioxidant power at N supply of 120 mg/L N. Controversially, Machado et al. [9] reported a significant decrease in the FRAP activity of spinach leaf-blade with increased N application. Additionally, Li et al. [46] showed that increasing N application reduced the FRAP value of leaf mustard. The antioxidant power of the IF was significantly increased when compared to 0 N supply; however, it did not significantly vary in activity in var. ‘Acadia’ fertilised with 30 to 120 mg/L N. Further, the antioxidant activity of the IF of ‘Acadia’ fertilised with 60, 120 and 150 mg/L N increased non-significantly. Although the var. ‘Crosstrek’ displayed a non-significant increase in antioxidant power as the N fertilisation rates increased from 30 to 120 mg/L in the intestinal fraction, the highest antioxidant power of the bioaccessible IF was observed with N supplies of 120 mg/L and 150 mg/L. On the other hand, N supply from 30 to 150 mg/L non-significantly increased the antioxidant power of the bioaccessible IF of var. ‘Traverse’ during *in vitro* digestion. 

The DPPH scavenging activities were higher in all three varieties fertilised with 90 mg/L N. However, the DPPH antioxidant scavenging activity in the bioaccessible intestinal fraction did not vary significantly concerning different rates of N supply, and a similar trend was observed with all three varieties. The ABTS scavenging activity of all three varieties increased with the increasing N supply rates. The ‘Acadia’ and ‘Traverse’ varieties showed the highest ABTS scavenging activity at 120 and 150 mg/L N supply, while var. ‘Crosstrek’ showed the highest ABTS scavenging activity at 90, 120 and 150 mg/L N supply. However, the rates of N fertilisation did not significantly affect the ABTS scavenging activity of the bioaccessible intestinal fraction during *in vitro* digestion.

#### 3.4.4. Multivariate Analysis 

**Figure 1:**
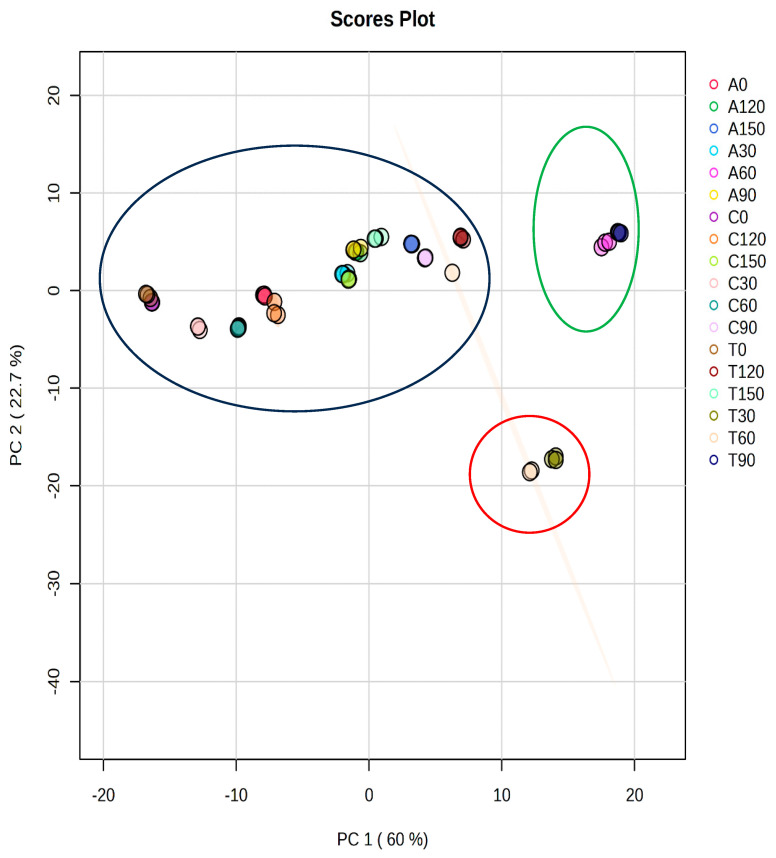
An unsupervised PCA scores plot of phenolic and carotenoid metabolites obtained via the HPLC-UV analysis of three spinach varieties and different nitrogen supplies. ‘Acadia’ (A), ‘Crosstrek’ (C) and ‘Traverse’ (T) were treated with different N concentrations including 0, 30, 60, 90, 120 and 150 mg/L.

Based on HPLC data obtained on phenolic and carotenoid compounds’ bioaccessibility, unsupervised PCA was conducted to show the impact of N application and varietal differences in baby spinach (Figure 1). PC1 explained 82.7% of the total variance (60.0% and 22.7%, respectively). Supplementary Figure S3 illustrates the loading of different phenolic and carotenoid metabolites in PC1 and PC2. The figure illustrates a systematic classification of N supply and spinach varieties based on their phenolic and carotenoid compounds’ bioaccessibility. The results showed that the phenolic and carotenoid bioaccessibility percentage in baby spinach varieties cultivated under different N supplies played a crucial role in their classification.

**Figure 2:**
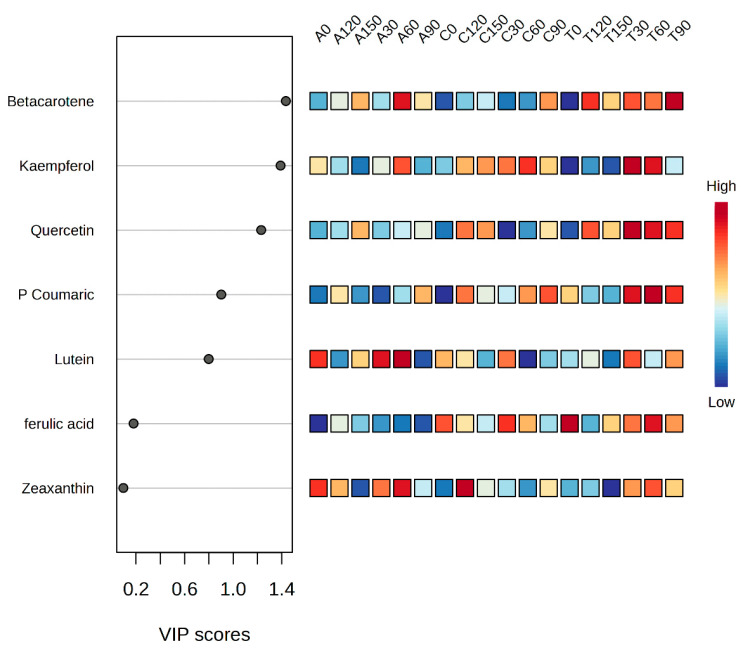
VIP scores in PLS-DA assigned to phenolic and carotenoid compounds found in baby spinach cultivars grown with different N concentration levels. ‘Acadia’ (A), ‘Crosstrek’ (C) and ‘Traverse’ (T) were treated with different N concentrations including 0, 30, 60, 90, 120 and 150 mg/L.

Good goodness-of-fit and predictability were both demonstrated by the PLS-DA model (R^2^ = 0.67), allowing for the prediction of metabolite changes based on the dataset. Principal component 1 (PC1) explained 52% of the total variation, and principal component 2 (PC2) explained 24.5%. Due to the distribution of samples, the PLS-DA plot showed two groups (Figure S3). The PLS-DA plot showed two distinct clusters of treatments (N supply and spinach variety) depending on the distribution of bioactive compounds’ bioaccessibility. ‘Traverse’, which received 30 or 90 mg/L N, was separated from the other cluster. This separation occurred because of the presence of β-carotene with loadings of PC1 at 0.953 and PC2 at −0.8455 (Figure S4). Bioactive compounds’ bioaccessibility with the highest VIP also received a characteristic score based on PLS-DA. Using the VIP score, the bioaccessibility of each bioactive compound was assessed to determine its contribution to cluster separation. VIP scores greater than 1 are used to detect the importance of a variable. The VIP values for the marker bioactive compounds’ bioaccessibility are presented in Figure 2. β-carotene, kaempferol and quercetin separated the ‘Traverse’ variety, which received 30 or 90 mg/L N, from the other cluster. A heat map structure was constructed by combining the concentrations of bioactive compounds and the N supply dosages for spinach varieties. 

**Figure 3:**
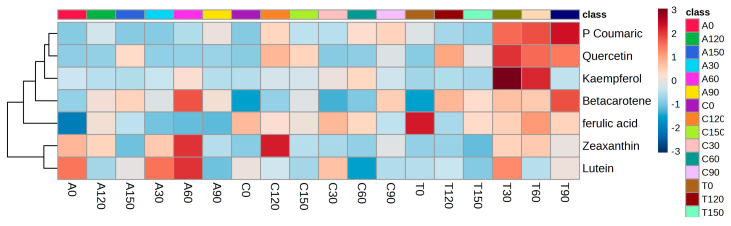
Heat map showing the phenolic and carotenoid compounds found in different baby spinach varieties grown with different nitrogen concentration levels. The rows represent the compounds, and the columns represent the spinach varieties at different N concentrations. The colours red and blue indicate high and low levels, respectively. ‘Acadia’ (A), ‘Crosstrek’ (C) and ‘Traverse’ (T) were treated with different N concentrations including 0, 30, 60, 90, 120 and 150 mg/L.

A heatmap (Figure 3) shows the over- and under-expression of specific bioactive compounds as a result of treatment interactions (N supply dosages for spinach varieties). Furthermore, heat maps shed light on patterns and groupings that might otherwise go unnoticed. Based on the heat map, var. ‘Transverse’ fertilised with 90 mg/L N supply showed the highest percentage of bioaccessibility of p-coumaric acid and beta carotene and moderately higher levels of quercetin from the IF.

This study showed that nitrogen fertilisation significantly affected the potential bioaccessibility of spinach phytochemicals. However, ‘Traverse’ with 90 mg/N mL increased the *in vitro* bioaccessibility of β-carotene, p-coumaric acid, quercetin and ferulic acid without compromising the yield. As suggested by Sęczyk et al. [15], our findings show that inadequate fertilisation led to dosage-dependent alterations in metabolism in spinach, which subsequently altered the matrix composition and influenced the bioaccessibility of different bioactive compounds. 

## 4. Conclusions

The morphology, colour properties and yield in three spinach varieties were significantly affected by nitrogen fertilisation. The study results suggest that nitrogen fertilisation is important for increasing the marketable yield and influencing the bioaccessibility of phenolic compounds, carotenoids and antioxidant activities. In addition to N supply, varietal differences play a major role in the bioaccessibility of phytochemicals in leafy vegetables like spinach. 

Additionally, different varieties showed different rates of nitrogen application for improving the bioaccessibility of p-coumaric, quercetin, ferulic acid and kaempferol during *in vitro* digestion. However, higher N levels affected the accumulation of different phenolic compounds. Although higher N application rates accumulated carotenoids in the leaves of the three spinach varieties, lower nitrogen application rates of 30, 60 and 90 mg/L N positively influenced the bioaccessibility of carotenoid components. For these three spinach varieties, the antioxidant activity of the bioaccessibility fraction was not affected by the N application. The varietal difference significantly played a role in determining the bioaccessibility of phenolic compounds and carotenoid components *in vitro*. By applying the appropriate N level during plant cultivation, these spinach varieties can be optimised for the bioaccessibility of their bioactive compounds without compromising the marketable yield. Therefore, applying the appropriate N level during cultivation and selecting suitable varieties or genotypes can help achieve the bioaccessibility of phenolic compounds and carotenoids without compromising the marketable yield.

## Figures and Tables

**Table 1:** Effect of nitrogen supply on the content and bioaccessibility of total phenols and selected phenolic compounds in three baby spinach cultivars.

Cultivar	Nitrogen Level (mg/L)	Undigested	Intestinal Fraction (IF)	Bioaccessibility (%)
TPC (mg/g)				
‘Acadia’	0	7.34 ± 0.15 ^h^	3.84 ± 0.24 ^de^	52.27 ± 3.32 ^cdef^
	30	7.77 ± 0.12 ^fg^	4.12 ± 0.63 ^cde^	52.99 ± 8.08 ^cdef^
	60	8.33 ± 0.26 ^bc^	3.534 ± 0.254 ^def^	42.41 ± 3.04 ^fg^
	90	8.09 ± 0.03 ^de^	3.713 ± 0.379 ^de^	45.88 ± 4.68 ^efg^
	120	7.94 ± 0.13 ^defg^	4.289 ± 0.561 ^bcd^	54.01 ± 7.06 ^bcdef^
	150	7.72 ± 0.032 ^g^	4.610 ± 0.447 ^bcd^	59.74 ± 5.79 ^bcde^
‘Crosstrek’	0	7.30 ± 0.03 ^h^	3.062 ± 0.241 ^ef^	41.92 ± 3.29 ^fg^
	30	7.83 ± 0.09 ^fg^	3.845 ± 0.611 ^de^	49.10 ± 7.81 ^def^
	60	8.45 ± 0.23 ^ab^	5.355 ± 0.377 ^ab^	63.39 ± 4.46 ^bcd^
	90	7.48 ± 0.20 ^h^	3.823 ± 0.057 ^de^	51.09 ± 0.77 ^cdef^
	120	7.72 ± 0.09 ^g^	5.270 ± 0.297 ^abc^	68.29 ± 3.85 ^b^
	150	7.72 ± 0.02 ^g^	6.412 ± 0.075 ^a^	83.09 ± 0.98 ^a^
‘Traverse’	0	7.81 ± 0.17 ^fg^	2.471 ± 0.356 ^f^	31.64 ± 4.56 ^g^
	30	8.02 ± 0.10 ^def^	3.521 ± 0.462 ^def^	43.92 ± 5.76 ^fg^
	60	8.62 ± 0.09 ^a^	4.591 ± 0.156 ^bcd^	53.29 ± 1.81 ^cdef^
	90	8.17 ± 0.03 ^bc^	3.901 ± 0.173 ^de^	47.76 ± 2.12 ^ef^
	120	8.11 ± 0.06 ^cde^	3.954 ± 0.083 ^de^	48.75 ± 1.03 ^def^
	150	7.92 ± 0.03 ^efg^	5.196 ± 0.581 ^bc^	65.58 ± 7.33 ^bc^
p-coumaric (µg/g)				
‘Acadia’	0	293.4 ± 1.24 ^a^	4.05 ± 0.02 ^d^	1.38 ± 0.01 ^i^
	30	217.2 ± 1.02 ^c^	2.95 ± 0.03 ^defg^	1.36 ± 0.01 ^i^
	60	152.2 ± 0.67 ^g^	3.70 ± 0.81 ^de^	2.43 ± 0.53 ^ghi^
	90	164.5 ± 0.84 ^f^	6.70 ± 0.02 ^c^	4.08 ± 0.01 ^de^
	120	55.9 ± 0.23 ^o^	1.86 ± 0.04 ^gh^	3.33 ± 0.06 ^efg^
	150	87.5 ± 0.43 ^j^	1.23 ± 0.02 ^h^	1.40 ± 0.03 ^i^
‘Crosstrek’	0	230.1 ± 0.48 ^b^	3.03 ± 0.02 ^def^	1.32 ± 0.01 ^i^
	30	215.8 ± 0.73 ^d^	5.63 ± 0.30 ^c^	2.61 ± 0.14 ^fgh^
	60	61.6 ± 1.07 ^n^	2.90 ± 0.02 ^efg^	4.72 ± 0.03 ^cd^
	90	184.2 ± 0.20 ^e^	9.92 ± 0.02 ^a^	5.39 ± 0.01 ^c^
	120	54.8 ± 0.93 ^o^	2.87 ± 0.46 ^efg^	5.24 ± 0.84 ^c^
	150	75.4 ± 0.1.71 ^l^	2.08 ± 0.02 ^fgh^	2.76 ± 0.02 ^fgh^
‘Traverse’	0	107.1 ± 0.27 ^i^	3.84 ± 0.91 ^de^	3.59 ± 0.85 ^def^
	30	119.2 ± 0.05 ^h^	10.43 ± 0.01 ^a^	8.75 ± 0.01 ^a^
	60	86.8 ± 0.14 ^j^	8.23 ± 0.58 ^b^	9.48 ± 0.67 ^a^
	90	75.0 ± 0.20 ^l^	5.35 ± 0.06 ^c^	7.13 ± 0.08 ^b^
	120	81.9 ± 0.71 ^k^	1.74 ± 0.49 ^h^	2.13 ± 0.60 ^hi^
	150	66.3 ± 0.62 ^m^	1.15 ± 0.01 ^h^	1.73 ± 0.01 ^hi^
Quercetin (µg/g)				
‘Acadia’	0	5687 ± 3.5 ^e^	21.05 ± 0.73 ^cd^	0.37 ± 0.01 ^g^
	30	4454 ± 1.4 ^f^	19.87 ± 0.09 ^cd^	0.45 ± 0.00 ^g^
	60	3950 ± 8.5 ^g^	23.47 ± 1.26 ^c^	0.59 ± 0.03 ^g^
	90	3340 ± 0.7 ^h^	22.86 ± 0.20 ^c^	0.68 ± 0.01 ^g^
	120	477 ± 0.7 ^l^	2.36 ± 3.11 ^e^	0.49 ± 0.65 ^g^
	150	297 ± 1.7 ^n^	12.67 ± 0.09 ^cde^	4.27 ± 0.03 ^e^
‘Crosstrek’	0	6931 ± 7.9 ^a^	17.59 ± 0.35 ^cd^	0.25 ± 0.00 ^g^
	30	6666 ± 0.3 ^b^	9.56 ± 0.42 ^de^	0.14 ± 0.01 ^g^
	60	5705 ± 4.1 ^d^	21.02 ± 0.15 ^cd^	0.37 ± 0.00 ^ef^
	90	428 ± 7.5 ^m^	11.96 ± 0.15 ^cde^	2.79 ± 0.03 ^f^
	120	204 ± 3.5 ^q^	12.23 ± 0.31 ^cde^	5.99 ± 0.15 ^d^
	150	261 ± 1.4 ^o^	12.39 ± 0.08 ^cde^	4.75 ± 0.03 ^e^
‘Traverse’	0	6621 ± 2.0 ^c^	15.02 ± 2.47 ^cde^	0.23 ± 0.04 ^g^
	30	574 ± 0.7 ^j^	60.22 ± 0.43 ^a^	10.49 ± 0.07 ^a^
	60	531 ± 0.71 ^k^	46.37 ± 17.41 ^b^	8.73 ± 3.28 ^b^
	90	596 ± 0.9 ^i^	49.41 ± 2.89 ^b^	8.29 ± 0.48 ^b^
	120	227 ± 1.6 ^p^	15.40 ± 0.49 ^cde^	6.78 ± 0.21 ^c^
	150	474 ± 0.1 ^j^	14.68 ± 0.27 ^cde^	3.10 ± 0.06 ^f^
Ferulic acid (µg/g)				
‘Acadia’	0	6908 ± 9.2 ^a^	37.93 ± 0.42 ^e^	0.55 ± 0.01 ^f^
	30	6592 ± 6.0 ^b^	72.53 ± 0.54 ^cd^	1.10 ± 0.01 ^defg^
	60	6232 ± 0.4 ^c^	65.88 ± 2.09 ^de^	1.06 ± 0.03 ^efg^
	90	5576 ± 4.6 ^g^	58.11 ± 0.30 ^de^	1.04 ± 0.00 ^fg^
	120	3732 ± 4.4 ^o^	64.93 ± 0.67 ^de^	1.74 ± 0.02 ^bcdef^
	150	4558 ± 9.3 ^l^	65.37 ± 0.11 ^de^	1.43 ± 0.00 ^cdef^
‘Crosstrek’	0	5352 ± 8.8 ^h^	112.22 ± 0.61 ^ab^	2.10 ± 0.01 ^abc^
	30	5818 ± 7.0 ^f^	122.81 ± 5.09 ^a^	2.11 ± 0.09 ^abc^
	60	6020 ± 2.9 ^d^	113.64 ± 56 ^ab^	1.89 ± 0.01 ^bcdef^
	90	5845 ± 8.6 ^e^	84.90 ± 0.73 ^bcd^	1.45 ± 0.01 ^bcdef^
	120	4999 ± 7.4 ^j^	89.00 ± 12.10 ^bcd^	1.78 ± 0.24 ^bcdef^
	150	4173 ± 1.9 ^n^	71.68 ± 0.84 ^cd^	1.72 ± 0.02 ^bcdef^
‘Traverse’	0	3566 ± 3.7 ^p^	105.77 ± 42.02 ^ab^	2.97 ± 1.18 ^a^
	30	5062 ± 4.8 ^i^	97.99 ± 0.73 ^abc^	1.94 ± 0.01 ^bcd^
	60	4516 ± 4.6 ^m^	104.62 ± 4.68 ^ab^	2.32 ± 0.10 ^ab^
	90	4513 ± 1.2 ^m^	86.85 ± 0.48 ^bcd^	1.92 ± 0.01 ^bcde^
	120	4930 ± 0.1 ^k^	65.65 ± 0.96 ^de^	1.33 ± 0.02 ^cdefg^
	150	3328 ± 1.8 ^q^	61.59 ± 0.24 ^de^	1.85 ± 0.01 ^bcdef^
Kaempferol (µg/g)				
‘Acadia’	0	211.6 ± 0.09 ^a^	7.55 ± 0.24 ^c^	3.57 ± 0.11 ^b^
	30	142.4 ± 0.18 ^f^	4.91 ± 0.03 ^c^	3.45 ± 0.02 ^b^
	60	149.7 ± 0.83 ^e^	9.83 ± 0.15 ^bc^	6.56 ± 0.10 ^b^
	90	149.8 ± 0.66 ^e^	3.60 ± 0.03 ^c^	2.41 ± 0.02 ^b^
	120	127.3 ± 1.14 ^g^	3.40 ± 0.02 ^c^	2.67 ± 0.01 ^b^
	150	190.3 ± 0.24 ^c^	4.33 ± 0.04 ^c^	2.28 ± 0.02 ^b^
‘Crosstrek’	0	74.0 ± 0.90 ^q^	1.80 ± 0.01 ^c^	2.44 ± 0.01 ^b^
	30	83.6 ± 0.53 ^o^	4.65 ± 0.22 ^c^	5.56 ± 0.27 ^b^
	60	86.6 ± 0.02 ^n^	6.68 ± 0.09 ^c^	7.72 ± 0.11 ^b^
	90	98.2 ± 0.38 ^l^	3.80 ± 0.03 ^c^	3.86 ± 0.03 ^b^
	120	119.4 ± 0.42 ^h^	4.96 ± 0.69 ^c^	4.16 ± 0.58 ^b^
	150	116.7 ± 0.82 ^i^	4.91 ± 0.06 ^c^	4.21 ± 0.05 ^b^
‘Traverse’	0	78.1 ± 0.14 ^p^	1.13 ± 0.04 ^c^	1.45 ± 0.05 ^b^
	30	108.2 ± 0.03 ^k^	25.86 ± 0.21 ^a^	23.90 ± 0.19 ^a^
	60	97.0 ± 0.53 ^m^	18.06 ± 12.91 ^ab^	18.62 ± 13.30 ^a^
	90	110.2 ± 0.38 ^j^	3.38 ± 0.05 ^c^	3.07 ± 0.05 ^b^
	120	195.9 ± 0.28 ^b^	4.52 ± 0.01 ^c^	2.30 ± 0.01 ^b^
	150	158.1 ± 0.12 ^d^	2.53 ± 0.02 ^c^	1.60 ± 0.01 ^b^

Data are means and standard deviation (n = 3). Means followed by the same letter within the column for each section are not significantly different (*p* < 0.05).

**Table 2:** Effect of nitrogen supply on the content and bioaccessibility of carotenoid compounds in three baby spinach cultivars.

Cultivar	Nitrogen Level (mg/L)	Undigested	Intestinal	Bioaccessibility (%)
TCC (mg/g)				
‘Acadia’	0	0.59 ± 0.00 ^hi^	0.14 ± 0.02 ^l^	23.90 ± 3.03 ^i^
	30	0.58 ± 0.01 ^i^	0.22 ± 0.01 ^k^	38.53 ± 1.48 ^h^
	60	0.63 ± 0.00 ^f^	0.59 ± 0.00 ^c^	93.60 ± 0.58 ^b^
	90	0.76 ± 0.00 ^b^	0.41 ± 0.00 ^g^	53.73 ± 0.25 ^g^
	120	0.78 ± 0.01 ^a^	0.58 ± 0.00 ^c^	74.65 ± 0.23 ^c^
	150	0.75 ± 0.00 ^b^	0.74 ± 0.01 ^a^	97.56 ± 0.77 ^a^
‘Crosstrek’	0	0.52 ± 0.02 ^k^	0.02 ± 0.00 ^p^	4.04 ± 0.73 ^m^
	30	0.55 ± 0.00 ^j^	0.05 ± 0.00 ^o^	9.64 ± 0.22 ^l^
	60	0.60 ± 0.00 ^gh^	0.08 ± 0.00 ^n^	12.98 ± 0.21 ^k^
	90	0.61 ± 0.00 ^g^	0.34 ± 0.00 ^i^	55.30 ± 0.57 ^fg^
	120	0.68 ± 0.01 ^de^	0.38 ± 0.00 ^h^	55.78 ± 0.18 ^f^
	150	0.70 ± 0.00 ^d^	0.68 ± 0.01 ^b^	97.38 ± 1.18 ^a^
‘Traverse’	0	0.52 ± 0.01 ^k^	0.02 ± 0.00 ^p^	3.87 ± 0.25 ^m^
	30	0.58 ± 0.00 ^i^	0.13 ± 0.00 ^m^	22.32 ± 0.29 ^j^
	60	0.59 ± 0.01 ^hi^	0.32 ± 0.00 ^j^	53.98 ± 0.39 ^g^
	90	0.64 ± 0.00 ^f^	0.42 ± 0.01 ^f^	66.54 ± 1.23 ^e^
	120	0.67 ± 0.01 ^e^	0.44 ± 0.01 ^e^	65.22 ± 0.96 ^e^
	150	0.74 ± 0.02 ^c^	0.54 ± 0.00 ^d^	72.86 ± 0.63 ^d^
LSD 0.05		0.01546	0.007963	1.648
CV%		1.5	1.4	2.0
Lutein (µg/g)				
‘Acadia’	0	4224 ± 2 ^o^	725.45 ± 0.71 ^m^	17.17 ± 0.02 ^b^
	30	7388 ± 8 ^l^	1274.17 ± 8.77 ^k^	17.25 ± 0.12 ^b^
	60	14,143 ± 9 ^k^	2835.45 ± 26.03 ^e^	20.05 ± 0.18 ^a^
	90	41,421 ± 10 ^e^	1960.91 ± 17.36 ^i^	4.73 ± 0.04 ^m^
	120	48,957 ± 5 ^d^	3249.75 ± 22.08 ^d^	6.64 ± 0.04 ^k^
	150	68,180 ± 16 ^a^	4602 ± 5.61 ^a^	9.55 ± 0.37 ^f^
‘Crosstrek’	0	3475 ± 4 ^q^	344.42 ± 2.44 ^p^	9.91 ± 0.07 ^e^
	30	3578 ± 6 ^p^	466.77 ± 1.91 ^o^	13.04 ± 0.05 ^d^
	60	25,380 ± 3 ^i^	601.83 ± 5.66 ^n^	2.37 ± 0.02 ^n^
	90	27,409 ± 0 ^h^	1992.74 ± 23.82 ^i^	7.27 ± 0.09 ^j^
	120	29,355 ± 6 ^g^	2653.77 ± 254.76 ^f^	9.03 ± 0.87 ^g^
	150	67,409 ± 8 ^b^	4509.80 ± 49.76 ^b^	6.69 ± 0.07 ^k^
‘Traverse’	0	4446 ± 5 ^n^	326.73 ± 3.69 ^p^	7.35 ± 0.08 ^ij^
	30	5482 ± 1 ^m^	880.01 ± 3.60 ^l^	16.05 ± 0.07 ^c^
	60	22,845 ± 17 ^j^	1711.69 ± 28.96 ^j^	7.49 ± 0.13 ^i^
	90	22,853 ± 0 ^j^	2294.33 ± 5.46 ^h^	10.04 ± 0.02 ^e^
	120	30,281 ± 0 ^f^	2484.93 ± 41.54 ^g^	8.21 ± 0.14 ^h^
	150	62,989 ± 5 ^c^	3427.59 ± 31.37 ^c^	5.44 ± 0.05 ^l^
LSD 0.05		8.553	38.24	0.2055
CV%		0.0	1.1	1.3
Beta-carotene (µg/g)				
‘Acadia’	0	5520 ± 1 ^n^	478.82 ± 3.07 ^n^	8.67 ± 0.06 ^l^
	30	7222 ± 2 ^m^	1098.44 ± 15.38 ^l^	15.21 ± 0.21 ^k^
	60	11,384 ± 4 ^h^	3981.27 ± 45.26 ^b^	34.97 ± 0.40 ^b^
	90	14,237 ± 2 ^g^	2509.91 ± 48.92 ^g^	17.63 ± 0.34 ^i^
	120	18,377 ± 2 ^d^	3231.47 ± 14.21 ^e^	17.58 ± 0.08 ^i^
	150	18,466 ± 3 ^c^	3912.12 ± 6.89 ^c^	21.19 ± 0.04 ^g^
‘Crosstrek’	0	4765 ± 5 ^q^	44.23 ± 0.71 ^p^	0.93 ± 0.01 ^o^
	30	4836 ± 3 ^p^	158.86 ± 1.66 ^o^	3.28 ± 0.03 ^n^
	60	8634 ± 1.8 ^l^	604.32 ± 6.98 ^m^	6.70 ± 0.08 ^m^
	90	8687 ± 2 ^k^	1897.66 ± 4.02 ^i^	21.84 ± 0.05 ^f^
	120	18,932 ± 1 ^b^	1650.80 ± 34.98 ^j^	8.72 ± 0.18 ^l^
	150	19,036 ± 8 ^a^	2975.48 6.64 ^f^	15.63 ± 0.03 ^j^
‘Traverse’	0	4655 ± 2 ^r^	40.06 ± 2.17 ^p^	0.86 ± 0.05 ^o^
	30	5136 ± 4 ^o^	1214.81 ± 9.08 ^k^	23.65 ± 0.18 ^d^
	60	9523 ± 2 ^j^	2134.69 ± 26.85 ^h^	22.42 ± 0.28 ^e^
	90	11,242 ± 3 ^i^	3970.24 ± 4.64 ^b^	35.32 ± 0.04 ^a^
	120	17,043 ± 3 ^f^	4212.31 ± 7.97 ^a^	24.72 ± 0.05 ^c^
	150	17,114 ± 1 ^e^	3309.84 ± 49.73 ^d^	19.34 ± 0.29 ^h^
LSD 0.05		5.356	38.32	0.2969
CV%		0.0	1.1	1.1
Zeaxanthin (µg/g)				
‘Acadia’	0	5441 ± 0 ^r^	82.76 ± 0.42 ^k^	1.52 ± 0.01 ^c^
	30	9966 ± 1 ^m^	135.06 ± 3.91 ^h^	1.35 ± 0.04 ^d^
	60	15,336 ± 1 ^k^	379.61 ± 6.32 ^b^	2.47 ± 0.04 ^b^
	90	27,988 ± 2 ^g^	156.47 ± 4.97 ^g^	0.56 ± 0.02 ^h^
	120	35,685 ± 4 ^f^	442.97 ± 0.51 ^a^	1.24 ± 0.00 ^e^
	150	47,654 ± 5 ^a^	94.7 ± 0.21 ^j^	0.15 ± 0.08 ^l^
‘Crosstrek’	0	7847 ± 1 ^p^	23.13 ± 0.23 ^m^	0.29 ± 0.00 ^k^
	30	9638 ± 1 ^n^	46.50 ± 0.27 ^l^	0.48 ± 0.00 ^i^
	60	13,956 ± 4 ^l^	48.02 ± 0.24 ^l^	0.34 ± 0.00 ^jk^
	90	25,474 ± 3 ^h^	219.92 ± 1.82 ^e^	0.86 ± 0.01 ^g^
	120	4415 ± 3 ^e^	129.7 ± 5.02 ^h^	2.62 ± 0.55 ^a^
	150	44,214 ± 3 ^d^	253.38 ± 0.76 ^c^	0.57 ± 0.00 ^h^
‘Traverse’	0	5939 ± 1 ^q^	20.98 ± 0.18 ^m^	0.35 ± 0.00 ^j^
	30	8061 ± 1 ^o^	102.52 ± 0.24 ^i^	1.27 ± 0.00 ^e^
	60	18,984 ± 1 ^j^	257.31 ± 1.30 ^c^	1.35 ± 0.01 ^d^
	90	24,775 ± 3 ^i^	229.74 ± 10.05 ^d^	0.93 ± 0.04 ^f^
	120	46,044 ± 11 ^c^	173.15 ± 1.58 ^f^	0.38 ± 0.00 ^j^
	150	46,554 ± 3 ^b^	47.71 ± 1.41 ^l^	0.10 ± 0.00 ^l^
LSD 0.05		4.288	5.753	0.05292
CV%		0.0	2.2	3.3

Data are means and standard deviation (n = 3). Means followed by the same letter within the column for each section are not significantly different (*p* < 0.05).

**Table 3:** Effect of nitrogen supply on the antioxidant activity and bioaccessibility in three baby spinach cultivars.

Cultivar	Nitrogen Level (mg/L)	Undigested	Intestinal
FRAP (mM/g)			
‘Acadia’	0	28.71 ± 1.53 ^g^	2.87 ± 0.13 ^e^
	30	34.98 ± 2.50 ^d^	4.51 ± 0.82 ^cde^
	60	38.93 ± 1.74 ^b^	7.49 ± 2.45 ^abc^
	90	38.76 ± 1.00 ^b^	4.51 ± 0.75 ^cde^
	120	39.37 ± 1.04 ^b^	6.87 ± 1.13 ^abcd^
	150	35.48 ± 0.50 ^cd^	8.43 ± 1.30 ^ab^
‘Crosstrek’	0	23.76 ± 0.76 ^i^	3.58 ± 0.10 ^de^
	30	25.71 ± 0.50 ^h^	4.00 ± 0.35 ^cde^
	60	29.76 ± 0.76 ^fg^	4.65 ± 0.52 ^cde^
	90	30.09 ± 0.76 ^f^	5.26 ± 0.58 ^bcde^
	120	36.48 ± 0.76 ^c^	5.84 ± 0.27 ^abcde^
	150	36.37 ± 0.29 ^c^	9.00 ± 0.59 ^a^
‘Traverse’	0	24.93 ± 1.04 ^hi^	3.11 ± 0.50 ^e^
	30	35.65 ± 0.76 ^cd^	5.50 ± 2.81 ^abcde^
	60	32.59 ± 0.75 ^e^	4.65 ± 1.17 ^cde^
	90	35.48 ± 1.32 ^cd^	5.86 ± 1.96 ^abcde^
	120	37.68 ± 0.76 ^a^	6.08 ± 0.65 ^abcde^
	150	35.65 ± 1.04 ^cd^	6.34 ± 0.50 ^abcde^
LSD 0.05			1.255
CV%			2.3
DPPH (IC_50_, mg/mL)			
‘Acadia’	0	1.90 ± 0.03 ^cdef^	2.27 ± 0.51 ^a^
	30	1.96 ± 1.96 ^efg^	2.54 ± 0.85 ^a^
	60	2.04 ± 0.02 ^gh^	1.83 ± 0.40 ^a^
	90	1.89 ± 0.03 ^cde^	2.49 ± 0.30 ^a^
	120	2.08 ± 0.06 ^hi^	3.68 ± 0.08 ^ab^
	150	2.69 ± 0.05 ^k^	4.79 ± 0.21 ^ab^
‘Crosstrek’	0	1.85 ± 0.04 ^cd^	4.91 ± 2.53 ^ab^
	30	1.93 ± 0.02 ^def^	3.99 ± 1.62 ^ab^
	60	1.95 ± 0.02 ^efg^	2.81 ± 0.49 ^a^
	90	1.83 ± 0.01 ^c^	5.42 ± 0.43 ^ab^
	120	2.16 ± 0.03 ^i^	5.36 ± 0.82 ^ab^
	150	2.29 ± 0.02 ^j^	7.82 ± 3.94 ^b^
‘Traverse’	0	1.52 ± 0.02 ^a^	3.22 ± 0.64 ^ab^
	30	1.67 ± 0.01 ^b^	3.95 ± 0.70 ^ab^
	60	1.84 ± 0.01 ^cd^	4.24 ± 0.82 ^ab^
	90	1.58 ± 0.07 ^ab^	2.59 ± 0.53 ^a^
	120	1.89 ± 0.02 ^cde^	3.25 ± 0.55 ^ab^
	150	1.99 ± 0.00 ^fgh^	3.39 ± 0.28 ^ab^
ABTS (IC_50_, mg/mL)			
‘Acadia’	0	2.00 ± 0.03 ^b^	2.28 ± 0.04 ^abc^
	30	3.37 ± 0.02 ^g^	2.23 ± 0.19 ^ab^
	60	3.73 ± 0.07 ^hi^	2.59 ± 0.15 ^abcd^
	90	2.59 ± 0.01 ^de^	3.00 ± 0.61 ^cd^
	120	2.30 ± 0.02 ^c^	2.84 ± 0.20 ^bcd^
	150	2.18 ± 0.05 ^c^	3.13 ± 0.23 ^d^
‘Crosstrek’	0	1.82 ± 0.03 ^a^	2.11 ± 0.03 ^ab^
	30	2.88 ± 0.08 ^f^	2.10 ± 0.03 ^ab^
	60	3.61 ± 0.03 ^h^	1.96 ± 0.08 ^a^
	90	2.51 ± 0.08 ^de^	1.99 ± 0.15 ^a^
	120	2.51 ± 0.06 ^de^	2.21 ± 0.25 ^ab^
	150	1.93 ± 0.06 ^ab^	2.29 ± 0.05 ^abc^
‘Traverse’	0	1.93 ± 0.03 ^ab^	2.52 ± 0.05 ^abcd^
	30	2.95 ± 0.06 ^f^	2.16 ± 0.05 ^ab^
	60	3.76 ± 0.05 ^i^	1.91 ± 0.25 ^a^
	90	2.64 ± 0.03 ^e^	2.09 ± 0.19 ^ab^
	120	2.46 ± 0.06 ^d^	2.23 ± 0.60 ^ab^
	150	2.18 ± 0.01 ^c^	2.57 ± 0.03 ^abcd^

Data are means and standard deviation (n = 3). Means followed by the same letter within the column for each section are not significantly different (*p* < 0.05).

## Data Availability

The original contributions presented in the study are included in the article/Supplementary Materials, further inquiries can be directed to the corresponding author.

## Supplementary Materials

The following supporting information can be downloaded at https://www.mdpi.com/article/10.3390/foods13172667/s1: Figure S1: In vitro digestion flow diagram. SSF: simulated salivary fluid; SGF: simulated gastric fluid; SIF: simulated intestinal fluid; Table S1: Average ambient temperature, relative humidity and photosynthetically active radiation (PAR) at TUT experimental site (greenhouse) for seasons 1 and 2; Table S2A: Two-way analysis of variance (ANOVA) for the effect of nitrogen and baby spinach varieties on leaf fresh mass for seasons 1 and 2. Table S2B: Effect of nitrogen concentration on leaf fresh mass of three baby spinach varieties for season 1 (S1) and season 2 (S2). Figure S2: Positive linear trend between leaf fresh mass and N-level concentrations in the three studied spinach varieties for A) season 1 and (B) season 2. Table S3A: Two-way analysis of variance for effect of nitrogen and baby spinach varieties on leaf colour for S1 and S2. Table S3B: Regression equations for leaf colour (L*a*b*) of three baby spinach varieties (‘Acadia’, ‘Crosstrek’ and ‘Traverse’) for season 1 and season 2. Table S3C: Effect of nitrogen concentration on leaf colour (L*a*b*) of three baby spinach varieties for season 1 (S1) and season 2 (S2). Table S4A: Two-way analysis of variance for effect of nitrogen and baby spinach varieties on leaf chlorophyll for S1 and S2. Table S4B: Effect of nitrogen concentration on leaf chlorophyll content in baby spinach varieties for seasons 1 and 2. Table S4C: Regression equations for leaf chlorophyll of three baby spinach varieties (V) (‘Acadia’, ‘Crosstrek’ and ‘Traverse’) for season 1 and season 2. Table S5A: Effect of spinach variety and applied N-level interaction on the total phenol (TPC) compounds in the intestinal fraction of three baby spinach varieties during in vitro digestion. Table S5B: Effect of spinach variety and applied N-level interaction on different phenolic compounds in the intestinal fraction of three baby spinach varieties during in vitro digestion. Table S6: Effect of spinach variety and applied N-level interaction on total carotenoid compounds in the intestinal fraction of three baby spinach varieties during in vitro digestion. Table S7: Effect of spinach variety and applied N-level interaction on antioxidant activities in the intestinal fraction of three baby spinach varieties during in vitro digestion. Figure S3: PLS-DA score plots generated from HPLC-UV analysis of intestinal phenolic and carotenoid metabolites from spinach varieties fertilised with varying levels of nitrogen. Figure S4: PLS-DA score plots of phenolic and carotenoid metabolites generated by HPLC-UV analysis showing the separation of two clusters.
