# Supplementary material for: The Effects of Nitrogen Application and Varietal Variation on the Product Quality and In Vitro Bioaccessibility of Bioactive Compounds of Baby Spinach Varieties Grown in a Soilless Growth Medium
Source: Foods. 2024 Aug 24;13(17):2667. doi: 10.3390/foods13172667 (PMC11394059; doi:10.3390/foods13172667)
Supplement: Supplementary file 1 [file foods-13-02667-s001.zip › foods-3150934-supplementary.pdf]

Supplementary Table S1. Average ambient temperature, relative humidity and photosynthetically active radiation (PAR) at TUT experimental site (greenhouse) for seasons 1 and 2.

Crop duration (Monthly)	Temperature (^{0}C)			Relative humidity (%)			PAR
	Max	Min	Mean	Max	Min	Mean	(µ mol $m^{-2}s^{-1}$)
Summer-Oct-22	32.75	15.7	27.4	80.1	24.2	69.68	904.5
22-Nov	30.58	13.01	25.3	78	19.9	62.29	902.5
22-Dec	34.86	17.02	29	94.8	28	78.2	905
Autumn-Mar-23	27.8	10.2	22.66	86	26.5	70.5	890
23-Apr	22.78	6.45	20.2	94	30	78.34	882

Max: Maximum; Min: Minimum

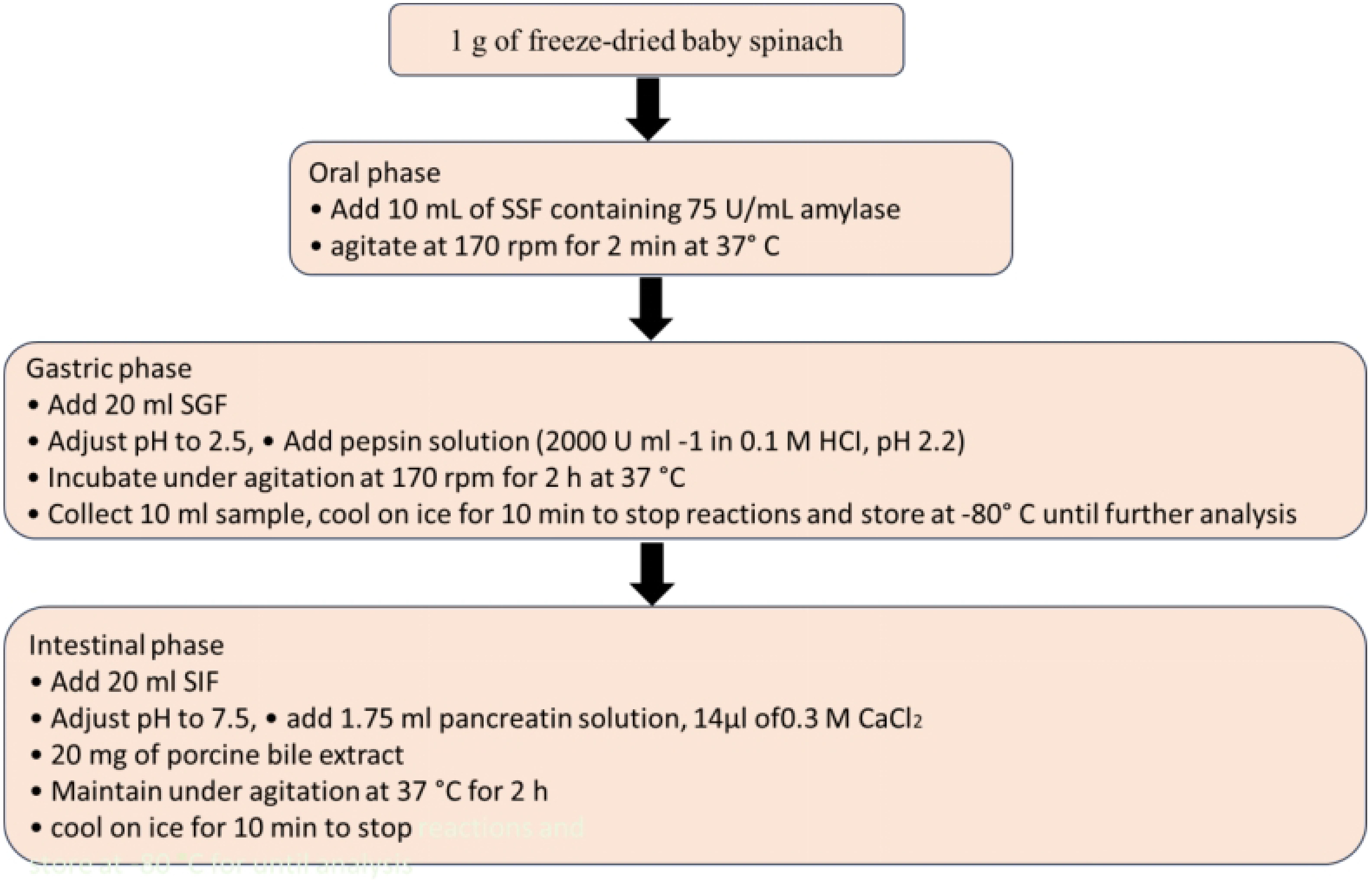

Supplementary Figure S1: *In vitro* digestion flow diagram. SSF: simulated salivary fluid; SGF: simulated gastric fluid; SIF: simulated intestinal fluid.

Table S2 A: Two-way analysis of variance (ANOVA) for the effect of nitrogen and baby spinach varieties on leaf fresh mass for seasons 1 and 2.

Source	Df	Fresh leaf mass	
		S1	S2
		MS	MS
Variety (V)	2	138207***	193933***
Nitrogen (N)	5	648423***	719775***
Vx N	10	25742***	32152***
Total	72		

*** *Significant at p<0.001.*
df, degrees of freedom; MS, mean squares; S1, season 1; 2, season 2

Table S2 B**:** Effect of nitrogen concentration on leaf fresh mass of three baby spinach varieties for season 1 (S1) and season 2 (S2).

Variety (V)	Nitrogen levels (mg/L)	Fresh leaf mass (g/m^2)	
		S1	S2
'Acadia'	0	108.1^{g}	109.0^{h}
	30	298.4^{f}	299.9^{g}
	60	397.9^{def}	377.1^{fg}
	90	557.9^{bc}	599.0^{cd}

	120	527.5^{cd}	540.6^{de}
	150	602.1^{bc}	609.8^{cd}
‘Crosstrek’	0	147.0^{g}	140.5^{h}
	30	287.7^{f}	283.4^{g}
	60	514.7^{cd}	531.9^{fg}
	90	614.1^{bc}	730.4^{b}
	120	856.3^{a}	921.4^{a}
	150	938.5^{a}	956.08^{a}
‘Traverse’	0	112.4^{g}	113.3^{h}
	30	310.4^{ef}	341.3^{fg}
	60	423.8^{de}	442.2^{ef}
	90	564.3^{bc}	554.8^{cd}
	120	605.5^{bc}	653.8^{bc}
	150	666.0^{b}	661.4^{bc}
LSD 0.05		124.38	110.22

[1] *Different letters within column represent significant differences at p≤0. 05.*

[2] *Values within column are expressed as means.*

[3] *Fisher’s protected t- test was adopted to determine or analyse the difference between means.*

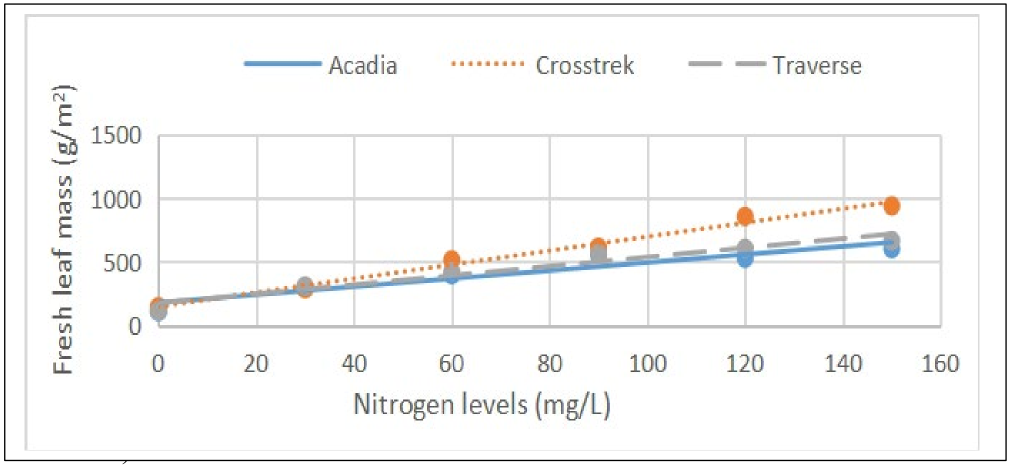

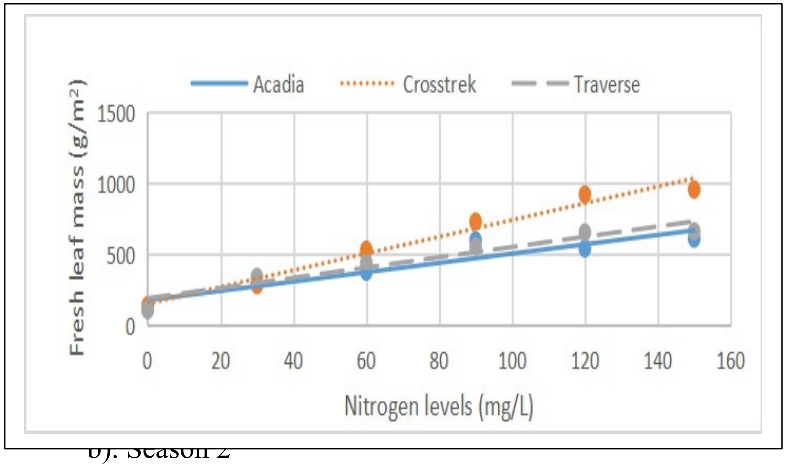

b). Season 2

Figure S2: Positive linear trend between leaf fresh mass and N-level concentrations in the three studied spinach varieties for A) season 1 and (B) season 2

Table S3 A: Two-way analysis of variance for effect of nitrogen and baby spinach varieties on leaf colour for S1 and S2.

**Source**	**df**	***L****		***a****		***b****	
		**MS**		**MS**		**MS**	
		**S1**	**S2**	**S1**	**S2**	**S1**	**S2**
Variety (V))	2	210.244***	106.934***	51.283***	46.124***	145.272***	203.059***
Nitrogen (N)	5	143.7024***	141.175***	9.472***	8.972**	249.534***	235.036***
V x N	10	36.4794***	45.873***	14.134***	6.407**	24.750***	29.992***

Total	71

***, ***, significant at p<0.01, p< 0.001.*

df, degrees of freedom; MS, mean squares.

Table S3 B: Regression equations for leaf colour *(L*a*b*)* of three baby spinach varieties ('Acadia', 'Crosstrek' and 'Traverse') for season 1 and season 2.

Leaf colour	Seasons (s1 & s2)	Regression equation	R^2
*a) L**			
· Acadia	**s1**	y= -0.00925x+43.846	0.99
	**s2**	y= -3.1268x+50.135	0.97
· Crosstrek	**s1**	y= -0.0083x+37.274	0.02
	**s2**	y= -0.4074x+39.803	0.07
· Traverse	**s1**	y= -0.0448x+45.263	0.35
	**s2**	y= -1.1645x+45.541	0.33
*b) a**			
· Acadia	**s1**	y= 0.019x+-11.233	0.67
	**s2**	y= 0.0212x-13.311	0.52
· Crosstrek	**s1**	y= -0.014x-11.06	0.2
	**s2**	y= -0.0133x-12.838	0.37
· Traverse	**s1**	y= -0.0212x-10.919	0.33
	**s2**	y= -0.0083x-13.704	0.2

*c) b**

·	Acadia	**s1**	y= -0.1086x+28.375	0.85
		**s2**	y= -0.1123x+30.339	0.88
·	Crosstrek	**s1**	y= -0.0427x+27.838	0.8
		**s2**	y= -0.0426x+30.177	0.61
·	Traverse	**s1**	y= -0.0832x+30.573	0.92
		**s2**	y= -0.0724x+32.351	0.86

Table S3C: Effect of nitrogen concentration on leaf colour (*L*a*b**) of three baby spinach varieties for season 1 (S1) and season 2 (S2).

Variety (V)	Nitrogen levels (mg/L)	**Leaf colour**					
		*L**		*a**		*b**	
		S1	S2	S1	S2	S1	S2
'Acadia'	0	43.93±1.075[b]	47.06±0.908[a]	-11.46±1.013[defg]	-12.048±1.303[bcde]	29.55±0.480[b]	32.18±1. 591[a]
	30	41.15±0.686[d]	45.27±0.874[ab]	-10.325±0.758[cde]	-13.881±2.981[defgh]	27.55±1.063[cd]	28.20±2.539[bc]

	60	38.45±0.592^{g}	39.15±1.971^{ef}	-9.573±3.387^{bc}	-12.685±0.755^{cdefg}	18.78±1.636^{i}	20.71±3.005^{f}
	90	34.47±0.914^{i}	36.84±2.826^{ghij}	-10.805±1.435^{cdef}	-11.974±1.058^{bcd}	15.63±0.822^{j}	17.66±2.611^{g}
	120	33.62±0.4435^{ij}	35.09±2.698^{ij}	-8.135±0.788^{a}	-9.171±0.312^{a}	14.95±0.420^{j}	16.55±0.312^{g}
	150	29.82±0.741^{k}	31.75±0.484^{k}	-8.529±0.409^{ab}	-10.563±0.332^{ab}	14.94±0.329^{j}	16.19±0.575^{g}
'Crosstrek'	0	39.55±0.777^{ef}	41.49±2.315^{cd}	-8.56±0.697^{ab}	-11.378±2.556^{bc}	27.05±1.316^{cd}	28.58±0.806^{b}
	30	38.67±0.665^{fg}	40.75±1.763^{de}	-13.225±0.656^{ijk}	-14.369±0.487^{fgh}	28.17±1.398^{bc}	32.01±2.493^{a}
	60	32.95±0.646^{j}	34.90±1.858^{j}	-13.453±0.296e^{jk}	-14.289±0.925^{fgh}	24.933±1.014^{e}	27.00±0.662^{bcd}
	90	33.40±0.920^{ij}	35.27±0.463^{hij}	-12.595±0.587^{ghij}	-14.2767±0.449^{fgh}	22.59±2.077^{fg}	24.42±2.091^{de}
	120	36.05±1.471^{h}	37.14±0.661^{fghi}	-13.07±0.577^{hijk}	-14.783±1.208^{h}	24.06±1.047^{ef}	24.065±1.047^{de}
	150	39.30±0.548^{fg}	40.72±0.169^{de}	-11.77±0.439^{efgh}	-13.929±0.317^{efgh}	21.01±0.630^{h}	26.37±1.047^{bcd}
'Traverse'	0	48.70±0.622^{a}	47.16±2.564^{a}	-10.428±0.820^{cde}	-12.45±2.229^{bcdef}	32.55±1.787^{a}	23.53±0.479^{e}

30	43.90±1.017[bc]	43.36±1.106[bc]	-10.057±0.606[cd]	-14.578±1.502[gh]	26.45±1.909[d]	34.04±0.479[a]
60	39.05±0.240[fg]	38.27±1.386e[fg]	-14.207±0.438[kl]	-14.86±0.938[h]	24.85±0.742[e]	28.53±0.888[bc]
90	36.52±0.602[h]	37.45±0,973[fgh]	-13.118±0.782[hijk]	-14.995±0.690[h]	22.07±1.179[gh]	26.58±2.425[bc]
120	42.77±0.580[c]	43.27±0.926[b]	-15.313±0.599[l]	-15.434±0.986[h]	21.42±1.135[gh]	25.92±2.207[cde]
150	40.47±0.512[de]	43.72± 1.600[bc]	-11.945±0.358[fghi]	-13.659±1.629[defgh]	18.65±0.705[i]	25.93±1.115[cde]
LSD 5%	1.08	2.33	1.46	1.92	1.5	3.07

[1] *Different letters within column represent significant differences at p≤0. 05.*

[2] *Values within column are expressed as means ± standard deviation.*

[3] *Fisher's protected t- test was adopted to determine or analyse the difference between means.*

Table S4A: Two-way analysis of variance for effect of nitrogen and baby spinach varieties on leaf chlorophyll for S1 and S2.

Source	Df	Leaf chlorophyll	
		MS S1	MS S2
Variety (V)	2	253.275***	297.858***
Nitrogen (N)	5	1682.166***	1641.171***

V x N	10	15.768***	16.044***
Total	71		

****Significant at p<0.001.*

df, degrees of freedom; MS, mean squares.

Table S4B: Effect of nitrogen concentration on leaf chlorophyll content in baby spinach varieties for seasons 1 and 2.

Variety (V)	Nitrogen concentration (mg/L)	Leaf chlorophyll (SPAD)	
		Season 1	Season 2
‘Acadia’	0	14.23±0.222[n]	17.18±0.465[m]
	30	19.21±0.299[j]	21.10±0.606[k]
	60	29.79±0.165[i]	37.02±1.040[g]
	90	34.54±0.269[f]	39.12±0.298[f]
	120	45.52±0.634[b]	47.98±0.499[b]
	150	46.16±0.171[a]	49.80±0.559[a]
‘Crosstrek’	0	11.43±0.171[q]	14.40±0.391[o]
	30	13.07±0.203[o]	18.05±0.420[l]
	60	26.41±0.349[k]	30.20±0.753[j]
	90	29.03±0.359[j]	31.55±0.794[i]
	120	32.32±0.288[g]	35.67±0.403[h]
	150	38.26±0.107[e]	40.08±0.171[e]

'Traverse'	0	11.87±0.281[q]	16.02±0.427[n]
	30	17.87±0.128[m]	20.77±0.680[k]
	60	29.14±0.206[j]	31.75±0.420[i]
	90	31.65±0.204[h]	37±0.572[g]
	120	39.72±0.358[d]	42.42±0.287[d]
	150	41.51±0.107[c]	44.35±0.603[c]
LSD 0.05		0.41	0.78

[1] Different letters within column represent significant differences at p≤0. 05.
[2] Values within column are expressed as means ± standard deviation.
[3] Fisher's protected t- test was adopted to determine or analyse the difference between means

Table S4C: Regression equations for leaf chlorophyll of three baby spinach varieties (V) ('Acadia', 'Crosstrek' and 'Traverse') for season 1 and season 2.

Variety (V)	Seasons (s1 & s2)	Regression equation	R^2
'Acadia'	**s1**	y= 0.2318x+14.19	0.97
	**s2**	y= 0.2341x+17.806	0.94
'Crosstrek'	**s1**	y= 0.1853x+11.188	0.94
	**s2**	y= 0.1739x+15.282	0.94

'Traverse'	**s1**	y= 0.206x+13.178	0.96
	**s2**	y= 0.2017x+16.924	0.96

Table S5A: Spinach variety and applied N level interaction on the total phenol (TPC) compounds in the intestinal fraction of three baby spinach varieties during *in vitro* digestion

Analysis of variance

Variate: TPC

Source of variation	d.f.	s.s.	m.s	v.r.	F pr
Variety	2	5.1210	2.5605	17.82	<.001
NITROGEN	5	27.7155	5.5431	38.57	<.001
Variety .NITROGEN	10	11.1822	1.1182	7.78	<.001
Residual	36	5.1737	0.1437		
Total	53	49.1924			

Table S5B: Spinach variety and applied N level interaction on different phenolic compounds in the intestinal fraction of three baby spinach varieties during *in vitro* digestion

Analysis of variance

Variate: Kaempferol

Source of variation	d.f.	s.s.	m.s	v.r.	F pr
Variety	2	362.68433	181.34216	5404.47	<.001
NITROGEN	5	1008.72097	201.7441	17137.56	<.001
Variety .NITROGEN	10	1226.85837	122.68584	10421.79	<.001
Residual	34	0.40025	0.01177		
Total	51	2243.45094			

Analysis of variance

Variate: Ferulic acid

Source of variation	d.f.	s.s.	m.s	v.r.	F pr
Variety	2	14057.326	7028.663	2101.19	<.001
NITROGEN	5	6758.007	1351.601	404.06	<.001
Variety .NITROGEN	10	5547.276	554.728	165.83	<.001
Residual	34	113.733	3.345		
Total	51	26257.834			

Analysis of variance

Variate: Quercetin

Source of variation	d.f.	s.s.	m.s	v.r.	F pr
Variety	2	4106.2426	2053.1213	3579.9	<.001
NITROGEN	5	3832.8423	766.5685	1336.62	<.001
Variety .NITROGEN	10	4242.4356	424.2436	739.73	<.001
Residual	34	19.4995	0.5735		
Total	51	10887.8739			

Analysis of variance

Variate: p-Coumaric acid

Source of variation	d.f.	s.s.	m.s	v.r.	F pr
Variety	2	44.0671	22.0336	167.69	<.001
NITROGEN	5	301.5241	60.3048	458.95	<.001
Variety .NITROGEN	10	4242.4356	11.2986	85.99	<.001
Residual	34	19.4995	0.1314		
Total	51	463.3080			

Table S6: Spinach variety and applied N level interaction of total carotenoid compounds in the intestinal fraction of three baby spinach varieties during *in vitro* digestion

Analysis of variance

Variate: Total carotenoids

Source of variation	d.f.	s.s.	m.s	v.r.	F pr
Variety	2	0.34804732	0.17402366	7540.34	<.001
NITROGEN	5	2.10171907	0.42034381	18213.25	<.001
Variety .NITROGEN	10	0.26420502	0.02642050	1144.78	<.001
Residual	35	0.00080777	0.00002308		
Total	52	2.67857043			

Analysis of variance

Variate: Lutein

Source of variation	d.f.	s.s.	m.s	v.r.	F pr
Variety	2	4892894.7	2446447.4	4616.82	<.001
NITROGEN	5	81843605.3	16368721.1	30890.30	<.001
Variety .NITROGEN	9	7604901.0	844989.0	1594.62	<.001
Residual	33	17486.6	529.9		
Total	49	72532545.7			

Analysis of variance

Variate: Zeaxanthin

Source of variation	d.f.	s.s.	m.s	v.r.	F pr
Variety	2	91653.85	45826.92	3812.29	<.001
NITROGEN	5	298554.21	59710.84	4967.28	<.001
Variety .NITROGEN	10	348452.61	34845.26	2898.74	<.001
Residual	34	408.71	12.02		
Total	51	734106.73			

Analysis of variance

Variate: Beta-carotene

Source of variation	d.f.	s.s.	m.s	v.r.	F pr
Variety	2	1.987E+07	9.935E+06	18552.34	<.001
NITROGEN	5	7.503E+07	1.501E+07	28022.27	<.001
Variety .NITROGEN	10	1.785E+07	1.785E+06	3333.90	<.001
Residual	36	1.928E+04	5.355E+02		
Total	53	1.128E+08			

Table S7: Spinach variety and applied N level interaction of Antioxidant activities in the intestinal fraction of three baby spinach varieties during *in vitro* digestion

Analysis of variance

Variate: FRAP

Source of variation	d.f.	s.s.	m.s	v.r.	F pr
Variety	2	2.685	1.343	18552.34	0.397
NITROGEN	5	113.303	22.661	28022.27	<.001
Variety .NITROGEN	10	33.938		3333.90	0.027
Residual	36	50.919		3.394	
Total	53	200.845		1.414	

Analysis of variance

Variate; DPPH

Source of variation	d.f.	s.s.	m.s	v.r.	F pr
Variety	2	54.044	27.022	20.06	<.001
NITROGEN	5	61.835	12.367	9.18	<.001
Variety .NITROGEN	10	47.224	4.722	3.50	0.003

Residual	35	47.158	1.347		
Total	52	176.652			

Analysis of variance

Variate: ABTS

Source of variation	d.f.	s.s.	m.s	v.r.	F pr
Variety	2	3.36324	1.68162	27.37	<.001
NITROGEN	5	1.66448	0.33290	5.42	<.001
Variety .NITROGEN	10	1.69390	0.16939	2.76	0.012
Residual	36	2.21153	0.06143		
Total	53	8.93316			

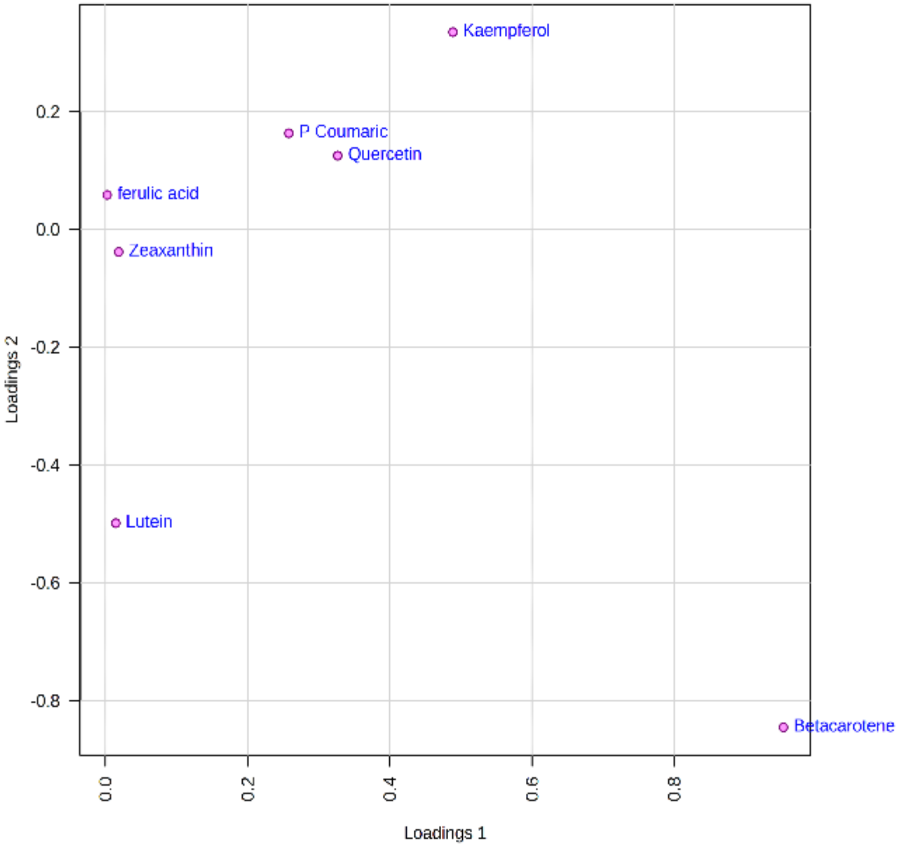

Figure S3: PLS-DA score plots generated from HPLC-UV analysis of intestinal phenolic and carotenoid metabolites from spinach varieties fertilized with varying levels of nitrogen

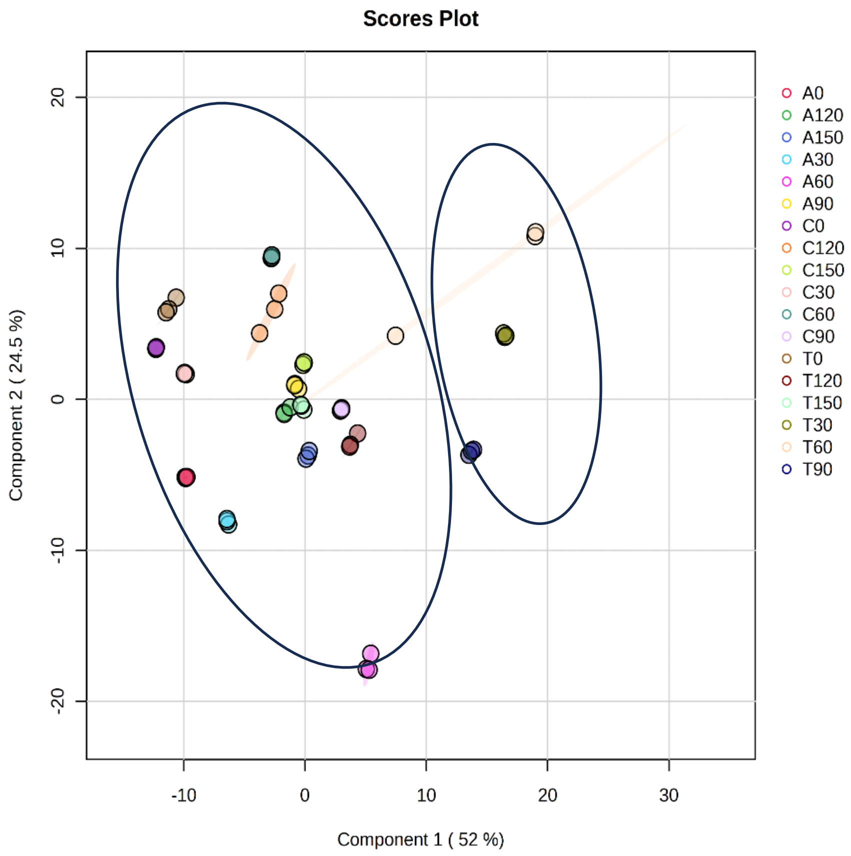

Figure S4: PLS-DA score plots of phenolic and carotenoids metabolites generated by HPLC-UV analysis showing the separation of two clusters.
